# Community-wide hackathons to identify central themes in single-cell multi-omics

**DOI:** 10.1186/s13059-021-02433-9

**Published:** 2021-08-05

**Authors:** Kim-Anh Lê Cao, Al J. Abadi, Emily F. Davis-Marcisak, Lauren Hsu, Arshi Arora, Alexis Coullomb, Atul Deshpande, Yuzhou Feng, Pratheepa Jeganathan, Melanie Loth, Chen Meng, Wancen Mu, Vera Pancaldi, Kris Sankaran, Dario Righelli, Amrit Singh, Joshua S. Sodicoff, Genevieve L. Stein-O’Brien, Ayshwarya Subramanian, Joshua D. Welch, Yue You, Ricard Argelaguet, Vincent J. Carey, Ruben Dries, Casey S. Greene, Susan Holmes, Michael I. Love, Matthew E. Ritchie, Guo-Cheng Yuan, Aedin C. Culhane, Elana Fertig

**Affiliations:** 1 Melbourne Integrative Genomics, School of Mathematics and Statistics, University of Melbourne, Melbourne, Australia; 2 McKusick-Nathans Institute of the Department of Genetic Medicine, Johns Hopkins School of Medicine, Baltimore, MD USA; 3 Data Science, Dana-Farber Cancer Institute, Boston, MA USA; 4 Department of Epidemiology and Biostatistics, Memorial Sloan Kettering Cancer Center, New York, NY USA; 5 Centre de Recherches en Cancérologie de Toulouse (INSERM), Université Paul Sabatier III, Toulouse, France; 6 Cancer Convergence Institute and Division of Quantitative Sciences, Department of Oncology, Sidney Kimmel Comprehensive Cancer Center, Johns Hopkins University School of Medicine, Baltimore, MD USA; 7 Department of Biostatistics, UNC, Chapel Hill, NC USA; 8 Barcelona Supercomputing Center, Barcelona, Spain; 9 Department of Statistics, University of Wisconsin, Madison, WI USA; 10 Department of Statistical Sciences, University of Padova, Padova, PD Italy; 11 Department of Pathology and Laboratory Medicine, University of British Columbia, Vancouver, BC Canada; 12 Department of Computational Medicine and Bioinformatics, University of Michigan, Ann Arbor, MI USA; 13 Department of Biomedical Engineering, University of Michigan, Ann Arbor, MI USA; 14 Department of Neuroscience, Johns Hopkins University, Baltimore, MD USA; 15 Klarman Cell Observatory, Broad Institute of MIT and Harvard, Cambridge, MA USA; 16 Department of Computer Science and Engineering, University of Michigan, Ann Arbor, MI USA; 17 Epigenetics and Development Division, The Walter and Eliza Hall Institute of Medical Research, University of Melbourne, Melbourne, Australia; 18 Epigenetics Programme, Babraham Institute, Cambridge, CB22 3AT UK; 19 Channing Division of Network Medicine, Brigham and Women’s Hospital, Harvard Medical School, Boston, MA USA; 20 Department of Hematology and Oncology, Boston Medical Center, Boston, MA USA; 21 Center for Health AI and Department of Biochemistry and Molecular Genetics, University of Colorado School of Medicine, Aurora, CO USA; 22 Department of Statistics, Stanford University, Stanford, CA USA; 23 School of Mathematics and Statistics, University of Melbourne, Melbourne, Australia; 24 Department of Genetics and Genomic Sciences, Charles Bronfman Institute for Personalized Medicine, Icahn School of Medicine at Mount Sinai, New York, NY USA; 25 Department of Biomedical Engineering, Johns Hopkins University School of Medicine, Baltimore, MD USA; 26 Department of Mathematics and Statistics, McMaster University, Hamilton, Canada; 27 Bavarian Center for Biomolecular Mass Spectrometry (BayBioMS), School of Life Sciences, Technical University of Munich, Munich, Germany; 28 Department of Genetics, UNC, Chapel Hill, NC USA; 29 Biostatistics, Harvard TH Chan School of Public Health, Boston, MA USA; 30 PROOF Centre of Excellence, Vancouver, BC Canada; 31 Kavli Neuroscience Discovery Institute, Johns Hopkins University, Baltimore, MD USA; 32 Department of Medical Biology, University of Melbourne, Melbourne, Australia; 33 Department of Computational Biomedicine, Boston University School of Medicine, Boston, MA USA; 34 Center for Regenerative Medicine (CReM), Boston University, Boston, MA USA; 35 Department of Applied Mathematics and Statistics, Johns Hopkins University Whiting School of Engineering, Baltimore, MD USA

## Introduction

Biological systems are fundamentally multi-scale, with mostly uncharacterized molecular pathways, cellular actions, and cellular communities that collectively give rise to their function. While one high-throughput measurement technology can resolve specific biological molecules, comprehensive characterization of biological systems can only be achieved by integration of multi-modal data types across molecular, cellular, spatial, and population scales. The integration of heterogeneous and complementary assays from multi-omics can reveal interactions between modalities that drive biological systems and processes. Recent advances in single-cell multi-omics technologies provide unprecedented opportunities for such multi-scale characterization but interpreting biological processes from these data requires parallel advances in novel computational techniques.

Advances in multi-omics technologies are creating tremendous new data resources and emerging atlas-based initiatives to uncover fundamental cellular biology. Single-cell multi-omics technologies have started to be developed only 6 years after single-cell sequencing. Their promise to the scientific community has been widely recognized, and they were even proclaimed the method of the year of 2019 to provide unique opportunities to characterize each cell at both spatial and molecular levels [1]. To date, these technologies have mostly focused on matched pairs of datasets such as mRNA-genome sequence, mRNA-DNA methylation, mRNA-chromatin accessibility, and mRNA-protein at the single-cell resolution, using assays such as scRNA-seq for transcriptomics, G&T-seq [2] and scTRIO-seq [3] for genomics (DNA and RNA), CITE-seq [4] for proteomics (surface protein and RNA), scNMT-seq [5] for epigenomics (DNA methylation and chromatin accessibility), to list a few [6]. These technologies provide what we refer to in this manuscript as multiple *modalities* of information. However, the rapid progress of technology development has outpaced the complementary computational advances necessary to analyze these data in an integrated fashion to uncover regulatory biology.

The goal of multi-modal single-cell data analysis is ultimately to explore relationships between data modalities, such as cell type-specific *cis* gene regulatory mechanisms observed between chromatin accessibility and gene expression. Computational methods for single-cell data integration have evolved from the extensive literature of multi-omics bulk data integration ﻿methods. These broadly fall into two categories. First, clusters can be built using pairwise distance matrices to identify common subgroups of features, such as cell types, in each data modality or between data modalities [7] that are subsequently input to cell type-specific network methods to infer regulatory mechanisms. Second, dimension reduction approaches can extract and combine latent components of global variance that are shared between data modalities [8], thereby learning novel cellular and molecular pathways associated with biological processes directly from the data. However, single-cell data differ in their resolution, size, scale, and sparsity that present new computational challenges not addressed in the algorithms developed for bulk multi-omics data. For example, the advent of spatial assays requires new adaptations of techniques from environmental statistics to infer cellular communities driving phenotypic fates in biological systems. In addition, one multi-omics dataset may require a breadth of analysis methods to uncover distinct regulatory processes.

Novel computational methods to analyze single-cell multi-omics data from these technologies are rapidly emerging. Ground truth is necessary to ensure analysis methods uncover accurate cell biology, but is missing to formulate the optimal models that underlie computational methods for multi-omics analysis. As a result, comprehensive assessment of new methods’ performance is often missing. Instead, the efficacy of these methods is usually assessed via visualization and biological and functional assessment of marker gene lists derived from prior biological knowledge. Quantitative comparisons between analysis methods are challenged by the lack of gold standard benchmarking datasets in the field and lack of biological ground truth. In fact, biological discovery of the regulatory processes that span molecular scales is an active area of biological research and a key motivation for generating multi-modal single-cell datasets. Often, conceptual advances to model innovative regulatory mechanisms make greater advances to multi-modal data analysis than do advances to raw performance metrics. Thus, benchmarking these techniques requires qualitative assessment supported through mechanistic experimental validation rather than the traditional quantitative assessment used in computational disciplines for methodological performance.

Collaborative community analysis of standardized datasets provides a transparent, reproducible, and reliable way to review the current state of the art in single-cell multi-modal data analysis [9]. In addition, enabling these analyses through open science on publicly available datasets can identify the range of computational challenges for the single-cell multi-omics community and catalyze the development of unforeseen algorithmic advances. Indeed, the wealth of biological knowledge that can be gleaned from independent analysis approaches can help identify not only common themes but also technology-specific challenges to be solved. To enable these efforts, we designed a series of three hackathons emblematic of current challenges that spanned spatial transcriptomics, spatial proteomics, and epigenomics. Although these tasks involved different biological processes and systems, we leveraged common analysis approaches, software infrastructures, and visualizations that are technology independent, while also demonstrating that some multi-omics approaches should also be biology or technology-specific. Datasets and analysis codes are publicly available on https://github.com/birsbiointegration as a resource to the community to expedite and advance the future of multi-omics data analysis.

This article articulates the needs for technologies, data, tools, and computational methods to model the multi-scale regulatory processes of biological systems in single-cell identified our three hackathon studies. It leverages these challenges to present a broad overview of the different types of analysis methods that can be currently applied to identify regulatory processes from multi-omics single-cell data sets and promising areas of future algorithmic development.

## Computational benchmarks are missing in cell biology

**Fig. 1:**
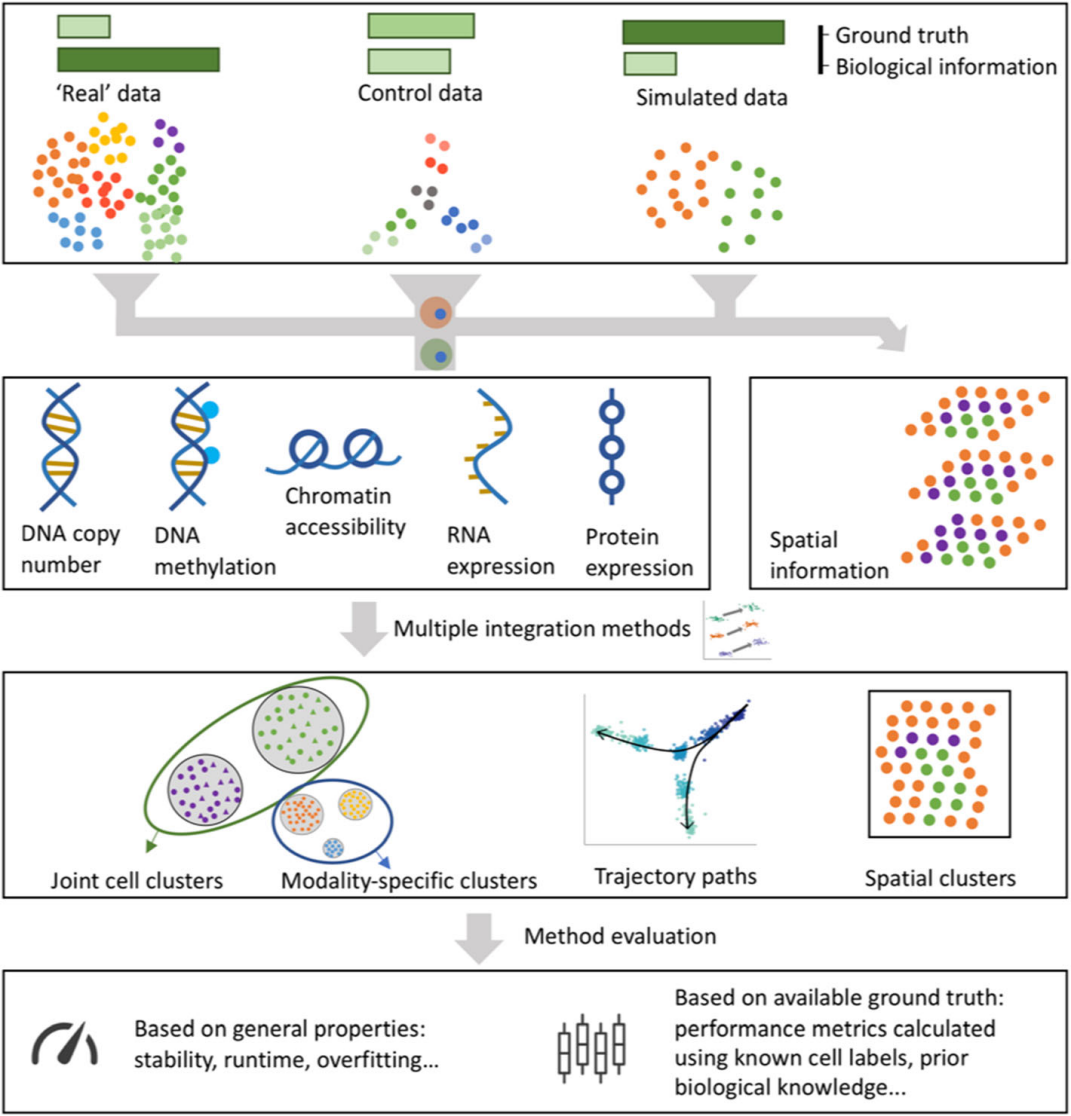
Systematic benchmarking of single-cell multi-omic analysis methods can involve experimental data (as per our hackathons), custom control datasets, where a known structure is imposed through the experimental design, or simulated data. The amount of biological signal and ground truth available varies considerably between these types of data. The generated multi-omics datasets are analyzed by competing methods and the results are compared using generic metrics (e.g., method stability, runtime, consistency via data splitting) or by considering the ground truth available (e.g., cell type labels or number of cell types)

In spite of the widespread demand for single-cell, multi-omics analysis methods, the computational biology community lacks standardized benchmarks to assess the performance and applicability of these methods. Benchmarking methods for multi-modal data is inherently difficult, as ground truth is rarely available. Many of the mechanisms through which molecular and cellular pathways interact across scales remain unknown. In the case of well-defined data integration tasks, ground truth can be introduced to assess computational performance by simulating high-throughput data in silico. Yet, the simulation of a realistic covariance structure across features is challenging and further complicated when modeling data across modalities in the context of data integration [10]. Common experimental designs to overcome these challenges involve creating artificial samples through the mixing of cells in known proportions [11–13] or creating dilution series to simulate variation in cell size [11, 14]. Simulating data is also popular and made more convenient through software such as the splatter R package [15] (Fig. 1). Still, these simulated data also rely on an underlying generative model that may introduce further biases based on the assumptions of that model and often cannot account for regulatory mechanisms that have yet to be discovered biologically.

The ideal benchmark datasets for multi-omics would be based on the biological reality of molecular and cellular networks, with full capacity to predict the biological impact of perturbations and temporal evolution. No universal benchmark data scheme may suit every combination of single-cell data modalities or biological problems. Instead, benchmark datasets should be established for commonly used combinations of modalities or technologies tailored to specific data integration tasks. For example, gene expression depends on gene regulatory element activity and thus requires an experimental design that must also account for spatial and temporal elements for a given cell. Therefore, defining a specific data integration task, and benchmarking the computational performance of the method for assessment relies on multi-modal data with specific study designs. These study designs should account for the biological dependencies between data modalities in sufficiently powered sample cohorts. The pervasive technical biases of high-throughput data require that the benchmark datasets and computational approaches also account for technical variability, leveraging block design and randomization to ensure that the data integration algorithm distinguishes intended regulatory processes from unintended technical variation.

## Assessing single-cell multi-omics analysis methods through hackathon studies

**Fig 2:**
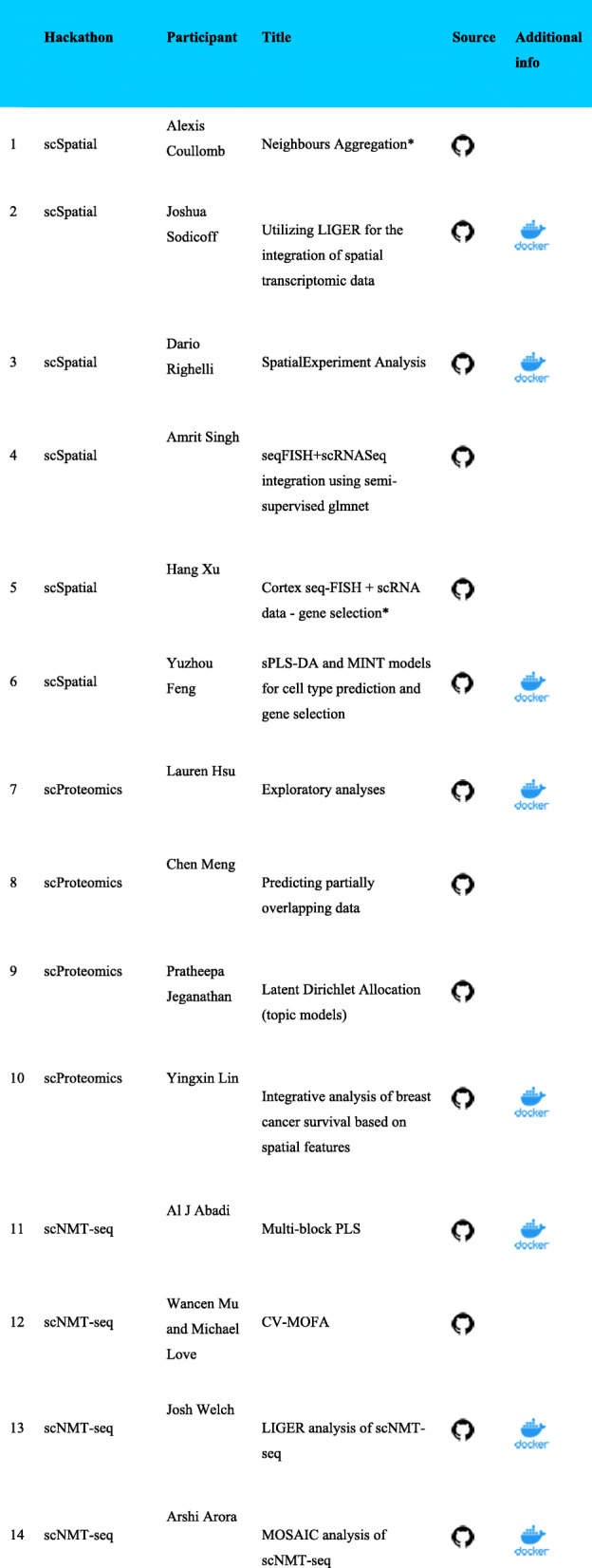
Vignettes for reproducible analyses are available at https://github.com/BIRSBiointegration/Hackathon/blob/master/analysis-vignettes.md. The hackathons analyses conducted in R were assembled into R packages as well as Docker containers. This allows reproducing the analysis environment in a seamless manner. Those conducted in Python marked with an asterisk (*) enabled automatic installation and deployment

One powerful means of engaging the community for comparison of multi-omics techniques is hackathon studies. These studies can leverage real data for benchmarking and defining challenges in multi-omics, even though ground truth is inherently unknown. Notably, the biology underlying a specific dataset can guide the question underlying the design of a specific multi-modal data challenge. Once a specified analysis task is identified, cross-validation within a study or across studies allows to assess whether solutions found by multi-modal methods generalize to held-out observations or held-out studies. We can also use subsampling on real-world data to assess the stability of the results (Fig. 2: 12, Additional file 1: Supplemental Notes S1 and S4). Finally, we can validate analysis approaches by benchmarking several algorithms and methods on the same dataset, allowing for open comparison of both standard and new methodologies. Although hackathons aim to standardize assessment of algorithm quality across research groups, the lack of ground truth of multi-omics data requires qualitative analysis of the inferred features across algorithms. Qualitative comparison of the classes of models used for analysis can also elucidate the range of biological questions and regulatory processes that can be determined from a single omics dataset. Most importantly, using community engagement in a hackathon enables us to define the specific problems in multi-modal data analysis that remain to be solved.

To review the latest computational frameworks for multi-modal analysis, we curated and designed hackathons for three datasets in single-cell multi-omics. Our studies focused on emblematic and emerging challenges in data integration across molecular and cellular scales, as well as emerging technologies. Our challenges ranged from the incorporation of cell spatial coordinates information (Hackathon 1 “Spatial transcriptomics” and Hackathon 2 “Spatial proteomics”), integration across different assays (Hackathon 3 “scNMT-seq”), and independent studies (Hackathon 2), epigenetic regulation of transcription (Hackathon 3), and cell type label prediction (all hackathons). The associated computational challenges are described in detail in Additional file 1: Supplemental Notes S1, S2, S3. In addition to reflecting different technologies, our hackathon studies were also designed to explore disparate challenges to multi-omics from different measurement technologies, while unified by the underlying problem of data integration. The complexity of the analysis challenges depended on the common information available across datasets, i.e., whether ‘omics included overlapping features but with various molecular resolution (Hackathon 1), were measured across independent studies or tissues (Hackathon 2), or were matched on the same cells (Hackathon 3).

All of our hackathon datasets are open access with complementary code for multi-omics analyses from our contributors on https://github.com/birsbiointegration. We leveraged and built on open frameworks to distribute the multi-omics data and share our analyses, using tools for continuous integration of changes to source codes (e.g., GitHub actions) and container snapshots of the analyses environments for reproducible analysis (Fig. 2). These open-source software efforts facilitate a community-level coordinated approach to support these validations through collaboration rather than duplication of effort between groups working on similar problems. A wide array of genomics frameworks for multi-platform single-cell data developed in R and Python are also available to the community (Table 1). We used the R/Bioconductor ecosystem for multi-omics to support our data structures, and the MultiAssayExperiment class from Bioconductor that provided not only efficient data storage but also enabled the implementation of further data processing and extraction of spatial information (Additional file 1: Supplemental Note S6). All participants provided vignettes describing their solutions to enable reproducible, open-source, and open development analysis (Fig. 2). In total, we pursued fourteen distinct analysis approaches that together illustrate technology-specific challenges as well as common themes for multi-omics analysis. Several challenges identified within each hackathon were addressed with a different set of methods that are listed in Table 2. The analyses conducted in each hackathon are detailed in the Additional file 1: Supplemental Notes S1, S2, S3.

**Table 1:** List of software for multi-modal single-cell analysis

Type	Name	Description
Matlab package	CytoMAP	CytoMAP: A Spatial Analysis Toolbox Reveals Features of Myeloid Cell Organization in Lymphoid Tissues
Matlab package	histoCAT	histoCAT: analysis of cell phenotypes and interactions in multiplex image cytometry data
Python library	PyTorch	General framework for deep learning
Python & R	TensorFlow	General framework for deep learning
Python package	SpaCell	SpaCell: integrating tissue morphology and spatial gene expression to predict disease cells
Python package	Scanpy	Python package for single-cell analysis
R data class	MultiAssayExperiment	unify multiple experiments
R data class	SpatialExperiment	SpatialExperiment: a collection of S4 classes for Spatial Data
R package	Giotto	Spatial transcriptomics
R package	cytomapper	cytomapper: Visualization of highly multiplexed imaging cytometry data in R
R package	Spaniel	Spaniel: analysis and interactive sharing of Spatial Transcriptomics data
R package	Seurat	R toolkit for single-cell genomics
R package	SpatialLIBD	Transcriptome-scale spatial gene expression in the human dorsolateral prefrontal cortex
R package	Cardinal	Cardinal: an R package for statistical analysis of mass spectrometry-based imaging experiments
R package	CoGAPS	scCoGAPS learns biologically meaningful latent spaces from sparse scRNA-Seq data
R package	projectR	ProjectR is a transfer learning framework to rapidly explore latent spaces across independent datasets
R package	SingleCellMultiModal	Serves multiple datasets obtained from GEO and other sources and represents them as MultiAssayExperiment objects
R scripts	SpatialAnalysis	Scripts for SpatialExperiment usage
Self-contained GUI	ST viewer	ST viewer: a tool for analysis and visualization of spatial transcriptomics datasets
Shiny app	Dynverse	A comparison of single-cell trajectory inference methods: towards more accurate and robust tools
R package	mixOmics	R toolkit for multivariate analysis of multi-modal data
R package	Corral	R package for dimension reduction and integration of single-cell data, using correspondence analysis
Python package	totalVI	A variational autoencoder (deep learning model) to integrate RNA and protein data from CITE-seq experiments
Python web application	ImJoy	Deep learning web interface
Python package	napari	Interactive big multi-dimensional 3D image viewer
Software	QuPath	Multiplex whole slide image analysis
Python package	Cytokit	Multiplex whole slide image analysis
Python package	cmIF	Multiplex whole slide image analysis
Software	Facetto	Multiplex whole slide image analysis, not available yet
Software, Python based	CellProfiler	Image analysis
Python library	Squidpy	Spatial single-cell analysis

**Table 2:** Different methods were used in the hackathons and are also available as reproducible vignettes

Common challenges	Tasks	Hackathon 1 (spatial transcriptomics)	Hackathon 2 (spatial proteomics)	Hackathon 3 (scNMT-seq)
**Pre-processing**	**Normalization & data transformation**	Data distribution checks (Fig. 2: 1, Fig. 2: 4) High Variable Genes selection (Fig. 2: 5)	Variance Stabilization Normalization [16] (Fig. 2: 8) Arcsinh transformation (Fig. 2: 9). Inverse transformation (Fig. 2: 9) Selection of patients (Fig. 2: 9)	Summaries of DNA measurements (input data provided in hackathon)
**Managing differences in scale**	**Data integration**	LIGER [17] (Fig. 2: 2) (sc) ComBat (Fig. 2: 4) (bulk) Projection methods MFA, sGCCA [18] (Fig. 2: 4^a^) (bulk) UMAP/tSNE (Fig. 2: 2) (sc)	Multiblock PCA [19] Weighting matrices based on their similarities: STATIS, MFA (Fig. 2: 8) (bulk^a^) Scale MIBI-TOF to the range of CyTOF values (Fig. 2: 9)	LIGER [17] (Fig. 2: 13) (sc) Projection method sGCCA [18] (Fig. 2: 11) (bulk) Multi Omics Supervised Integrative Clustering with weights (Fig. 2: 14) (bulk)
**Overlap**	**Cell overlap** (features not matching)			**Dimension reduction and projection methods:** LIGER [17] (Fig. 2: 13) (sc) sGCCA [18] (Fig. 2: 11) (bulk)
	**Partial feature overlap** (cells not matching)		**Imputation:** Direct inversion with latent variables Optimal transport to predict protein expression (Fig. 2: 10) K-nearest neighbor averaging (Fig. 2: 9) **No imputation:** Biological Network Interaction^a^	
	**Partial cell overlap** (features not matching)		Multiblock PCA [19] (Fig. 2: 8^a^)	
	**No cell overlap** (complete feature overlap)		Transfer cell type label with Random Forest (Fig. 2: 7)	LIGER [17] (Fig. 2: 13)
	**No cell overlap** (partial feature overlap)		Topic modeling to predict cell spatial co-location or spatial expression (Fig. 2: 9, partial feature overlap)	
	**No overlap**		RLQ^a^ [20]	
**Generic approaches**	**Classification & feature selection**	Backward selection with SVM (Fig. 2: 1) Self-training ENet (Fig. 2: 4) Balanced error rate (Fig. 2: 1) Fig. 2: 4) Recursive Feature Elimination (Fig. 2: 5) (all bulk)		Multi Omics Supervised Integrative Clustering (Fig. 2: 14) (bulk) Lasso penalization in regression-type models (bulk)
	**Cell type prediction**	Projection with LIGER [17] (Fig. 2: 2) SVM (Fig. 2: 1, Fig. 2: 5) ssEnet (Fig. 2: 4) (all bulk)		
	**Spatial analysis**	Hidden Markov random field Voronoi tesselation (Fig. 2: 1) (bulk)	Spatial autocorrelation with Moran’s Index (Fig. 2: 7, Fig. 2: 10) Selection of spatial discriminative features: Moran’s Index, NN correlation, Cell type, interaction composition, L function (Fig. 2: 10) (all bulk)	
	**Inclusion of additional information**		Survival prediction: Cox regression based on spatial features (Fig. 2: 10)	Include annotated hypersensitive sites index to anchor new/unseen data from DNase-seq, (sc)ATAC-seq, scNMT-seq, for de novo peak calling (bulk^a^)

^a^indicates that the method was not applied on the hackathon data, “bulk” indicates the method was originally developed for bulk omics, “sc” indicates the method was specifically developed for single-cell data, other methods are generic

### Hackathon-specific challenges

#### Hackathon 1: spatial transcriptomics and integration of scRNA-seq with seqFISH

The first hackathon focused on the integration of spatial transcriptomic profiling data with non-spatial scRNA-seq data. While spatial approaches are gaining popularity, they often provide lower molecular resolution than non-spatial methods. Strategies to integrate these data hold the promise to enhance the molecular resolution of spatially resolved profiling. This hackathon included spatial transcriptional data of 125 genes for 1597 single cells from seqFISH with corresponding non-spatial whole-transcriptome profiling of 1723 cells from the mouse visual cortex [21]. These datasets share measurements for 113 genes. The first challenge was to predict cell types in the seqFISH data, based on the putative cell types learnt from the scRNA-seq data, and determine a minimal number of genes necessary for data integration. The second challenge questioned whether gene signatures of cellular co-localization were preserved in the non-spatial transcriptional data.

In the first challenge, we explored several strategies to assign the most likely cell types to single cells in the seqFISH dataset based on information obtained from the scRNA-seq dataset (the latter being considered as a training data set). We used supervised and semi-supervised methods with feature selection, including support vector machines, sparse Partial Least Squares Discriminant Analysis (sPLS-DA) [22], and generalized linear models with elastic net penalty. Unsupervised integrative methods based on non-negative matrix factorization (NMF) [23] were also investigated. As ground truth, we considered the predicted cell types based on the original study from [24] that integrated the seqFISH and scRNA-seq data. Overall, our analyses achieved a prediction accuracy greater than 80%. This challenge highlighted typical issues encountered when conducting statistical learning from similar data types (here gene expression) measured using different assays (scRNA-seq and seqFISH) on different cells. First, training the classifier model required an already established ground truth. Second, the prediction assessment from the seqFISH data was limited by the lack of biological knowledge, the non-targeted nature of the genes sequenced—as those genes are not necessarily characteristics of those cell types, and the exploratory nature of our analyses. Third, the type of classifier (linear or non-linear, supervised, or semi-supervised) and the gene selection strategies were also found to influence the performance of the methods. Finally, the evaluation of the methods required an adequate choice of metrics to account for cell type imbalance inherent to the study.

In the second challenge, we sought to transfer spatial information obtained from the seqFISH dataset to that of the scRNA-seq dataset. We built spatial networks from cells’ positions in the seqFISH dataset by Delaunay triangulation [25]. The clusters of cells obtained from the spatial data did not necessarily overlap with specific cell types, suggesting that the spatial dimension cannot be captured from gene expression data only. In addition, we were unable to extract combinatorial spatial patterns directly from scRNA-seq data, even though previous studies have demonstrated cellular mapping between gene expression profiles and known spatial locations [26, 27]. Thus, we faced both technological and analytical challenges that will require careful benchmarking in the near future.

#### Hackathon 2: cross-platform and cross-study integration with spatial proteomics

The second hackathon focused on an integrative data analysis across studies and platforms with limited overlap in proteins between the two datasets. It included spatial proteomics matched with non-spatial data. Here we dealt with a typical scenario in clinical omics whereby datasets are obtained on different tissues using related but different platforms, studying the same disease. This hackathon contained two breast cancer cohorts. The first cohort included 143 subjects with 73 proteins profiled with single-cell proteomics mass cytometry (CyTOF) [28] and the second cohort 41 patients with 36 spatial in situ proteins profiled from Multiplexed Ion Beam Imaging (MIBI) [29]. A total of 20 proteins were assayed in both studies, with 6 patients in the CyTOF dataset and all 41 patients in the MIBI dataset of the triple-negative subtype of breast cancer. The main challenge was to predict cell labels and cell compositions from one dataset to the other when proteomics data are partially overlapping from different patients with similar phenotypes, in a cross-platform and cross-study setting.

Two main challenges emerged. The first challenge investigated whether analytical methods could integrate partially overlapping proteomics data collected on different patients with similar phenotypes, and whether measurements from one technology (MIBI spatial location and expression of proteins) could be transferred and used to predict information in the second technology (spatial expression patterns of proteins measured on CyTOF). Several semi-supervised and supervised algorithms were applied to transfer cell labels and cell compositions from one dataset to the other, including random forests [30] and entropic regularization optimal transport [31, 32]. The analyses highlighted a critical lack of methods for cell type assignment, classification, or extraction of differentially expressed proteins for targeted proteomics. In particular, we identified an urgent need for a unifying map between cells present in different datasets, and for annotation resources to provide quality metrics or priors of protein cell type markers. One solution would be to construct protein expression atlases across different studies to support cell type classification.

The second challenge explored the added value of spatial technologies to uncover information about immune cell populations in breast cancer beyond cell composition. K-nearest neighbor graph, topic models [33], and graph-based neighborhood measures were used, demonstrating the prognostic potential of spatial single-cell proteomics data. This challenge highlighted the need to develop new spatial measures specifically for single-cell spatial proteomics data.

#### Hackathon 3: scNMT-seq experiments and integration of RNA and DNA features on the same cells

The third hackathon explored multi-modal integration of data of different molecular modalities from the same cells. This study leveraged single-cell epigenetics data to investigate how genetic and epigenetic alterations to DNA drive the transcriptional regulation underlying cellular state transitions. Specifically, this third hackathon included scNMT-seq data from 826 cells with concurrent DNA methylation, chromatin accessibility, and RNA expression from the same cells during mouse embryonic development, spanning post-implantation and early gastrulation [34]. This hackathon presented the specific challenge of identifying associations between disparate molecular components where data sets differ in scale, size, and noise levels in integrative frameworks.

After defining different types of genomic contexts (e.g., promoters, enhancers), we conducted integrative analyses of five data modalities (Additional file 1: Figure S3A, gene expression, promoter, gene body, CGI and DHS methylation) using methods based on distance measures, NMF, and PLS. We assessed the ability of these methods to cluster cells based on their developmental stages. We additionally used data splitting to assess whether methods trained on one part of the scNMT-seq dataset had similar model performance on held-out cells (Additional file 1: Supplemental Note S4 and Figure S5). Our analyses showed that different data sets led to different clustering performance. Thus, identifying appropriate data sets to explain a phenotype (here developmental stage) is critical for integrative analysis.

In a second challenge, we examined the effect of imputing the numerous missing values in DNA methylation using methods such as nearest neighbor averaging. We found that clustering was improved when using imputed data, rather than dealing with methods (such as PLS) that handle missing values internally. Thus, more methodological developments for missing value imputation would be highly beneficial for the analysis of single-cell methylation data.

### Common challenges across hackathons

#### Choice of pre-processing approach

Due to the established impact of pre-processing on genomics analysis, we used our hackathons to assess the effect of normalization and data transformation (e.g., variance stabilization, arcsinh or inverse transformation in Hackathon 2), preliminary feature selection (mostly based on highly variable genes, Hackathon 1), or feature summarization (Hackathon 3). The best way to pre-process emerging data remains a challenge when there is a lack of ground truth. We used visualization of step-by-step transformations to clarify how certain methods fit models or reduce data dimensionality. These visualizations can often be very specialized (e.g., goodness of fit QQ-plots or rootograms, mean-variance fitting plots), but serve as intermediary checks to understand seemingly black box analytical processes. We also recommend applying different analyses to different input data and comparing the final results both from a numerical and biological perspective.

Pre-processing may also include how we define molecular units for each data modality. RNA-seq has well-defined units and IDs (e.g., transcript names), but other assays may need to be summarized at different genomic scales (e.g., gene promoters, exons, introns, or gene bodies, see Hackathon 3). Tools that compute summaries at different scales and different overlaps between signal (e.g., ATAC-seq peaks) and genomic annotation can address those challenges (e.g., R/GenomicRanges [35]). Finally, another challenge is that observations of different modalities may not be directly comparable: for instance, gene expression may be measured from individual cells in scRNA-seq, but spatial transcriptomics may have a finer (sub-cellular) or coarser (multi-cellular) resolution. Deconvolution methods of multi-cellular spots signal are currently emerging to address this issue [36].

#### Managing heterogeneity across datasets

**Fig. 3:**
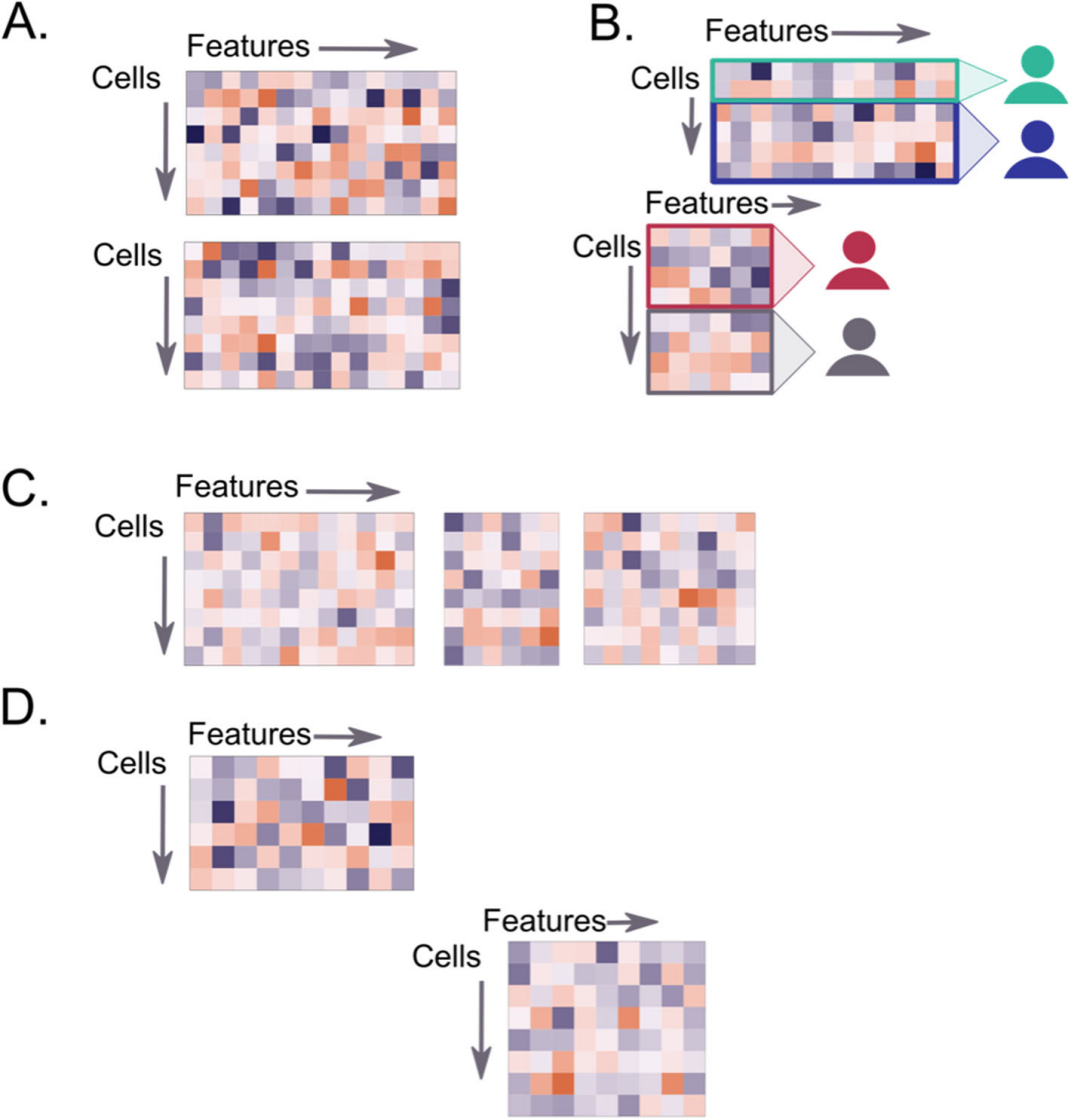
Common challenge in data integration: Addressing partial overlap of information across cells or features. **A** Overlap of features but not cells (e.g., Hackathon 1 where we considered the overlap of genes between seqFISH and scRNA-seq data). **B** Partial overlap of features but no overlap of cells (e.g., Hackathon 2 that required data imputation or cell type prediction, with different patients for each set of cells and only some proteins overlapping). **C** Overlap of cells across assays, but no overlap of features (e.g., Hackathon 3). **D** Lack of overlap between cells and features (the so-called fourth corner problem in Hackathon 2)

Differences in data set sizes and the specific molecular features measured in the assays constitute a major challenge for data integration. One solution to manage size was to reduce data to a common set of features (genes for Hackathon 1, or proteins for Hackathon 2, Fig. 3A) or via feature selection, either embedded in the methods (Hackathon 1) or ad hoc with highly variable features (Hackathon 3). Another alternative was to use projection-based methods that can manage differences in sizes across datasets (Hackathon 3).

Differences in data scale may result in one dataset contributing to either too much variation or noise during data integration. Existing approaches we have tested across all hackathons offered further improvement in the analyses, but there is still room for new techniques to be further developed to either (re)scale, remove batch effects (here due to systematic differences between platforms), or weighting specific data sets (e.g., [37] proposed by one of our participants).

Multiple domains of knowledge can be combined easily if there is a common coordinate system, as is the case in geospatial analyses. In both Hackathons 1 and 2, a spatial dimension was already naturally available, where we could leverage spatial statistics methods to quantify spatial effects. In these studies, similarity between expression profiles and inferred clusters could be tested and easily understood in the spatial context. Thus, for spatial data, layers of information can be mapped to the natural coordinate system in the same way a geographic information system framework incorporates them to identify a “consensus space” that provides a common reference system, as we further discuss in Additional file 1: Supplemental Note S5.

#### Managing lack of information overlap

The degree of feature or cell overlap between datasets varied dramatically within each hackathon. Intuitively, it is necessary that at least one type of overlap, whether on the features (Fig. 3A) or cells (Fig. 3C), is present to integrate information across disparate modalities. The field has made progress in developing methods to integrate data sets that match the same cells (Hackathon 3), especially based on dimension reduction techniques (e.g., NMF, or PLS, see Hackathons 1 and 3). However, when there is no cell overlap (spatial Hackathons 1 and 2), imputation methods are required to predict gene, protein, or spatial expression values (Fig. 3B). Methods ranged from nearest neighbors, latent variables, or optimal transport with some approaches that can also be used to predict cell types. When there is a complete lack of overlap between cells or features—the so-called fourth corner (Hackathon 2, Fig. 3D), one solution is to rely on (common) phenotypes of the cells to create some sort of overlap of information. We anticipate that this scenario will be avoided once technological progress and an increase in data availability is achieved [38].

### Interpretation of results

The analyses from each hackathon emphasized that regardless of the common difficulties faced by our participants, there was no “one method fits all” for multi-omics integration. An equally important complement to the diverse computational methods used to solve multi-omics analysis problems rests in the biological interpretation of their solutions. The high-dimensional nature of single-cell data already poses a challenge to interpretation and is further confounded by the often higher dimension resulting from concatenating datasets across molecular scales with multi-omics technologies. Interpretation hinges on the analytical methods selected for a given dataset. Some methods used in the hackathons and summarized in Table 2 aimed to predict a clearly defined outcome (e.g., cell labels). Supervised analyses often provide easier interpretations, as one can easily rank the covariates and contiguous data in terms of their predictive potential (Hackathon 1). However, when data are collected without the availability of a clear response (e.g., survival time, tumor size, cell growth) using multiple different technologies, data integration requires organizing patterns that enable interpretation.

Unsupervised analyses are widely applied to single-cell datasets to provide lower dimensional representations that facilitate interpretation and even latent variables that can reflect biological processes in the system. Low-dimensional representation of single-cell multi-omics data often requires additional contiguous data, such as spatial coordinates to capture higher-level cellular structure or prognostics (Hackathons 1 and 2). Clustering is often used as an unsupervised method that can use latent variables—for example using a categorical variable such as cell type, which was not directly measured on the data but enables simple interpretations [39] (Hackathon 3). Unfortunately, biological phenomena are often not as clear-cut. During clustering, over separating data by forcing the data into types only provides a static description when the variation should often be along a continuum. Indeed, although a latent factor can be a useful first approximation, the development of cells and their fate is a dynamic process. Thus, we recommend referring back to the original data that enabled interpretation of the cell trajectories: in our case, where the underlying latent variable of interest is expressed along a gradient of development (e.g., pseudo-time, disease progression).

**Fig. 4:**
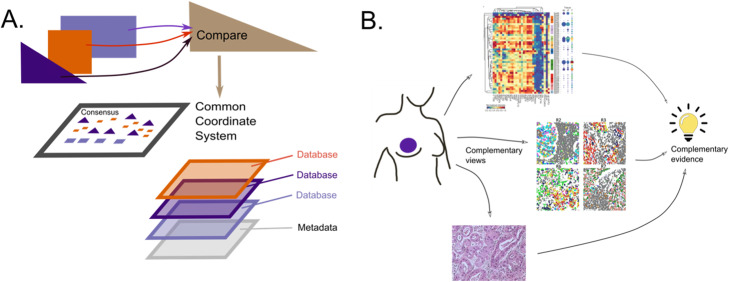
**A** Schematic diagram of stages of interpretation and integration of data sources. **B** Integrative analysis across multiple modes of data results in complementary evidence, allowing stronger conclusions, an instance of Cardinal Newman’s principle: “Supposes a thesis (e.g., the guilt of an accused man) is supported by a great deal of circumstantial evidence of different forms, but in agreement with each other; then even if each piece of evidence is in itself insufficient to produce any strong belief, the thesis is decisively strengthened by their joint effect.”

While methods to automatically interpret low-dimensional representations remains an open question, even in absence of biological annotation latent variables represent a rich anchor for many multi-modal methods and can often be useful in highlighting what the modalities have in “common” and how they differ, as highlighted in Hackathon 3. Disparate sources of evidence, or in this case, data from different technologies, are more compelling than many replicates of the same technology. Thus, if different technologies allow a consensus on underlying latent variables, this information is worth retaining. The commonalities are well understood in the case of classical multivariate factor analysis, where the data are decomposed into common and unique components [40]. A schematic summary of the different stages in interpretation is provided in Fig. 4).

### Improving interpretation

Information from external databases can be incorporated into the final output to improve interpretation (Fig. 4A). However, biological interpretation is often limited to the integration of contiguous information available from metadata or from exterior sources such as Gene Ontologies, Biomart [41], Kegg, Human Cell Atlas (HCA), or within software systems. As the high-dimensional nature of single-cell data can allow computational algorithms to have multiple solutions of similar accuracy, redundant biological knowledge can be enlightening. By providing information on the extreme points in a map or “brushing a map” with respect to known gene expression features, one can delineate orientations and clusters. As an example, it is only through coloring trajectories on UMAPs of selected genes that allows us to see the dynamics of cell-state transitions, similar to the principle behind the interactive brushing illustrated in Fig. 4B.

Interpretation of complex and rich data often relies on visualization strategies that use color, leading to difficulties in perceiving patterns for a substantial proportion of the population with color vision deficiencies and leading to different data interpretations between individuals. We recommend presenting accessible scientific information using colorblind-friendly visualizations and palettes [42–45] with a limit of 10 colors. Additional hatched areas or point shapes can also reduce the dependence on colors. The inclusion of a self-standing caption accompanying figures can guide the reader’s perception of the images and would greatly benefit broader data accessibility.

Finally, spanning all of these interpretation challenges is a central communication barrier between data analysts and the community of practitioners who do not have the same vocabulary or background. Many tools are used as black boxes where users do not have a clear understanding of the statistical or mathematical principles underpinning the methods. We recommend to the community the establishment of a clear glossary of terms, and how we are using those terms to improve communication. For example, many synonyms for multi-modal data exist and some have nuances, as we have collated in Table 3. Understanding the relationship between methods described by different teams is essential. Data scientists often start by categorizing methods first; thus, it is useful to present a dichotomy of methods and their underlying properties for biology collaborators.

**Table 3:** Glossary of terms

Consensus term	Related terms	Description	Citation
Network	Graph, adjacency matrix	A set of *nodes*, representing objects of interest, linked by *edges*, representing specific relationships between nodes.	[46]
Node	Vertex	Element of interest in a network and linked to other nodes. For example: people, cells, proteins or genes. Nodes can have several properties called *attributes* like cell type or position.	[46]
Edge	Link	The relationship between 2 nodes in a network. For example: friendship in social networks, cells in contact in a spatial network, or gene-gene interactions in a gene regulatory network.	[46]
Concordant	Common	Agreement between multiple modalities with respect to feature/variable selection and correlation of latent factors.	[47, 48]
Consistent	Coherent, self-consistent, within-study evaluation	Similar performance obtained from applying methods for multi-modal data during multiple rounds of data splitting.	[49]
Contributions	Variable weights, loadings, eigenvector, axis, direction, dimension, coefficients, slopes	Contributions of the original variables in constructing the components.	[50, 51]
Latent factors	Variates, scores, projections, components, latent/hidden/unobserved variables/factors	Weighted linear combinations of the original variables.	[50, 51]
Multi-modal	Multiview, multiway arrays, multi-modal, multidomain, multiblock, multitable, multi-omics, multi-source data analysis methods, N-integration	Methods pertaining to the analysis of multiple data matrices for the same set of observations.	[50, 52, 53]
Conjoint analysis	P-integration, meta-analysis, multigroup data analysis	Methods pertaining to the analysis of multiple data matrices for the same set of variables.	[50, 51, 54]
Variable	Feature	A measurable quantity that describes an observation’s attributes. Variables from different modalities include age, sex, gene or protein abundance, single nucleotide variants, operational taxonomic units, pixel intensity *etc.*	[46]
Biomarker	Marker	A variable that is associated with normal or disease processes, or responses to exposures, or interventions. Any change in this variable is also associated with a change in the associated clinical outcome. These variables may be used for diagnostic, monitoring, Pharmacodynamic responses. Examples include LDL cholesterol, CD4 counts, hemoglobin A1C.	[55]
Panel	Biomarker panel, biomarker signature	A subset of the originally measured variables that are determined to be associated with the outcome or response variable. This may be determined using statistical inference, feature selection methods, or machine/statistical learning.	[56, 57]
Observation	Sample, observation, array	A single entity belonging to a larger grouping. Examples include patients, subjects, participants, cells, biological sample, and usually the unit of observation on which the variables are measured	[46]

## Discussion

Our article highlights the power of hackathons to both inform and develop new analysis methods to capture the complex, multi-scale nature of biological datasets from high-throughput data modalities. Notably, our hackathon studies were specifically designed to span state-of-the-art multi-omics challenges to map the epigenetic, molecular, and cellular interactions across time and sample populations. Single-cell measurements spanning molecular modalities can inherently simplify the challenge of linking disparate biological scales, but layering new sets of molecular measurements increases the complexity of the analyses to interpret these data. The computational needs hinge on the underlying biological question being asked as well as on the characteristics of the data themselves.

In our analyses, different modeling considerations had to be made for multi-modal integration, as highlighted in Hackathons 1 and 3 (matching on the same genes, or cells) and Hackathon 2 (partially unmatched measurements). Our participants chose a wide range of approaches for each case study, common challenges were encountered, and common types of analyses were applied. Some analytical methods derived from bulk RNA-seq literature were able to answer the biological question posed in our hackathons, spanning from data integration, to cell type prediction, or spatial analysis. Some of the methods developed specifically for single-cell data did not necessarily perform well in our real case studies. Data heterogeneity and lack of overlap between data sets constitute the most important challenges for multi omics single-cell data integration.

Through these hackathons, we identified several common analysis themes spanning algorithmic advances, interpretation, benchmarking, and software infrastructure necessary for biological interpretation. All hackathons required methods for dealing with data quality, data loss from summarization, timing variances between and within omics layers, and batch effects. These represent the necessary challenges to overcome in the coming years, along with efficient and insightful data visualization strategies to infer regulatory relationships between different omics.

Technologies to profile biological systems at single-cell resolution and across molecular scales are advancing at an unprecedented pace. Analytically, these advances require the computational community to pursue research that can first enable analyses tailored to specific biological features or measurement technology, and second, that can scale and adapt to these rapid advances. Our hackathons highlighted current technologies for spatial molecular profiling. The two technologies used in this study both have limited molecular resolution. Therefore, multi-platform data combining the spatial molecular data from either seqFISH, MIBI, or imaging mass cytometry require integration of complementary data from other single-cell technologies to provide both high spatial and molecular resolution. We note that additional technologies, such as slide-seq [58] and Visium from 10X Genomics produce spatially resolved molecular measurements approaching measurements of the whole transcriptome, but lack the fine spatial resolution of imaging technologies. As such, emerging technologies still require further multi-platform data integration for comprehensive analysis. Hackathon 3 did not include spatially resolved data but highlighted the potential of further inference of gene regulation through concurrent profiling of RNA, methylation, and chromatin state. Technological advances for multi-omics spatial data and epigenetics data are rapidly advancing and becoming increasingly available through Nanostring, 10X Genomics, Akoya Biosciences, and others. New research-level technological advances enable three-dimensional spatial molecular profiling [59]. Other technologies are currently expanding to allow for temporally resolved profiling [60] or ATAC and gene expression on matched cells (chromium single-cell multiome). Integration strategies aware of these future directions and the mathematical challenges that span technologies will be most adept at advancing biological knowledge: this was the primary aim of our hackathons.

The implementation of novel analysis tools requires further software ecosystems, including Bioconductor [61], Biopython, and toolkits such as Scanpy [62], Seurat [63], or Giotto [64], in which users can implement their analysis approaches, while anticipating stable and adaptive data structures that are applicable for these emerging technologies. The size of these emerging datasets, particularly in the context of their application to atlas projects (e.g., the Human Tumor Atlas Network [65], Human Cell Atlas [66], Allen Brain Initiative, Brain Initiative Cell Census Network, or ENCODE/Roadmap/4D nucleome, to cite a few), are key examples that computational efficiency and scalability of these implementations are becoming ever more critical.

In addition to new technologies, we wish to emphasize that arising multi-omics analysis methods can support the generation of new data sources to resolve the multi-scale nature of biological systems. For example, our hackathons posed the scNMT-seq data (Hackathon 3) and spatial molecular datasets (Hackathons 1 and 2) as distinct challenges for data integration. However, integration of matched datasets between these spatial and epigenetic profiling techniques could further resolve the dependence of cell type and cellular interactions of regulatory networks. By embedding prior biological knowledge as rules in the analysis approaches, additional sources of data can generate a new representation of a biological system. For example, curated regulatory networks from databases such as KEGG, Biocarta, GO, TRANSFAC, reactome, or MSigDB provide commonly used frameworks for this prior knowledge. These gene regulatory networks must be extended to map the impact of cellular context on transcriptional regulation that are being uncovered by emerging single-cell atlases. The regulatory networks and dynamic features captured in single-cell data also provide the potential for techniques to predict molecular and cellular states, catalyzing new areas of research.

## Supplementary Information

**Additional file 1:S1.** Case study for spatial transcriptomics: integration of scRNA-seq + seqFISH. **S2.** Case study for cross-study and cross-platform analysis: spatial proteomics. **S3.** Case study for epigenetic regulation: scNMT-seq. **S4.** Further considerations on benchmarking. **S5.** Further considerations on results interpretation. **S6.** Further considerations on software.
