# Author Correction: Community-wide hackathons to identify central themes in single-cell multi-omics

**DOI:** 10.1186/s13059-021-02468-y

**Published:** 2021-08-25

**Authors:** Kim-Anh Lê Cao, Al J. Abadi, Emily F. Davis-Marcisak, Lauren Hsu, Arshi Arora, Alexis Coullomb, Atul Deshpande, Yuzhou Feng, Pratheepa Jeganathan, Melanie Loth, Chen Meng, Wancen Mu, Vera Pancaldi, Kris Sankaran, Dario Righelli, Amrit Singh, Joshua S. Sodicoff, Genevieve L. Stein-O’Brien, Ayshwarya Subramanian, Joshua D. Welch, Yue You, Ricard Argelaguet, Vincent J. Carey, Ruben Dries, Casey S. Greene, Susan Holmes, Michael I. Love, Matthew E. Ritchie, Guo-Cheng Yuan, Aedin C. Culhane, Elana Fertig

**Affiliations:** 1 Melbourne Integrative Genomics, School of Mathematics and Statistics, University of Melbourne, Melbourne, Australia; 2 McKusick-Nathans Institute of the Department of Genetic Medicine, Johns Hopkins School of Medicine, Baltimore, MD USA; 3 Data Science, Dana-Farber Cancer Institute, Boston, MA USA; 4 Biostatistics, Harvard TH Chan School of Public Health, Boston, MA USA; 5 Department of Epidemiology and Biostatistics, Memorial Sloan Kettering Cancer Center, New York, NY USA; 6 Centre de Recherches en Cancérologie de Toulouse (INSERM), Université Paul Sabatier III, Toulouse, France; 7 Cancer Convergence Institute and Division of Quantitative Sciences, Department of Oncology, Sidney Kimmel Comprehensive Cancer Center, Johns Hopkins University School of Medicine, Baltimore, MD USA; 8 Department of Mathematics and Statistics, McMaster University, Hamilton, Canada; 9 Bavarian Center for Biomolecular Mass Spectrometry (BayBioMS), School of Life Sciences, Technical University of Munich, Munich, Germany; 10 Department of Biostatistics, UNC, Chapel Hill, NC USA; 11 Barcelona Supercomputing Center, Barcelona, Spain; 12 Department of Statistics, University of Wisconsin, Madison, WI USA; 13 Department of Statistical Sciences, University of Padova, Padova, PD Italy; 14 Department of Pathology and Laboratory Medicine, University of British Columbia, Vancouver, BC Canada; 15 PROOF Centre of Excellence, Vancouver, BC Canada; 16 Department of Computational Medicine and Bioinformatics, University of Michigan, Ann Arbor, MI USA; 17 Department of Biomedical Engineering, University of Michigan, Ann Arbor, MI USA; 18 Department of Neuroscience, Johns Hopkins University, Baltimore, MD USA; 19 Kavli Neuroscience Discovery Institute, Johns Hopkins University, Baltimore, MD USA; 20 Klarman Cell Observatory, Broad Institute of MIT and Harvard, Cambridge, MA USA; 21 Department of Computer Science and Engineering, University of Michigan, Ann Arbor, MI USA; 22 Epigenetics and Development Division, The Walter and Eliza Hall Institute of Medical Research, University of Melbourne, Melbourne, Australia; 23 Department of Medical Biology, University of Melbourne, Melbourne, Australia; 24 Epigenetics Programme, Babraham Institute, Cambridge, CB22 3AT UK; 25 Channing Division of Network Medicine, Brigham and Women’s Hospital, Harvard Medical School, Boston, MA USA; 26 Department of Hematology and Oncology, Boston Medical Center, Boston, MA USA; 27 Department of Computational Biomedicine, Boston University School of Medicine, Boston, MA USA; 28 Center for Regenerative Medicine (CReM), Boston University, Boston, MA USA; 29 Center for Health AI and Department of Biochemistry and Molecular Genetics, University of Colorado School of Medicine, Aurora, CO USA; 30 Department of Statistics, Stanford University, Stanford, CA USA; 31 Department of Genetics, UNC, Chapel Hill, NC USA; 32 School of Mathematics and Statistics, University of Melbourne, Melbourne, Australia; 33 Department of Genetics and Genomic Sciences, Charles Bronfman Institute for Personalized Medicine, Icahn School of Medicine at Mount Sinai, New York, NY USA; 34 Department of Biomedical Engineering, Johns Hopkins University School of Medicine, Baltimore, MD USA; 35 Department of Applied Mathematics and Statistics, Johns Hopkins University Whiting School of Engineering, Baltimore, MD USA

**Correction to: Genome Biol 22, 220 (2021)**

**https://doi.org/10.1186/s13059-021-02433-9**

Following publication of the original article [1], the authors identified that author Dario Righelli had erroneously been omitted from the author panel. The author group above has been updated and the original article [1] has been corrected.
