# Supplementary material for: Community-wide hackathons to identify central themes in single-cell multi-omics
Source: Genome Biol. 2021 Aug 5;22:220. doi: 10.1186/s13059-021-02433-9 (PMC8340473; doi:10.1186/s13059-021-02433-9)
Supplement: Supplementary file 1 — Additional file 1:S1. Case study for spatial transcriptomics: integration of scRNA-seq + seqFISH. S2. Case study for cross-study and cross-platform analysis: spatial proteomics. S3. Case study for epigenetic regulation: scNMT-seq. S4. Further considerations on benchmarking. S5. Further considerations on results interpretation. S6. Further considerations on software. [file 13059_2021_2433_MOESM1_ESM.docx]

# Additional file 1

## **S1. Case study for spatial transcriptomics: integration of scRNA-seq + seqFISH**

### **Overview and biological question**

This hackathon aimed to leverage the complementary strengths of sequencing and imaging-based single-cell transcriptomic profiling by using computational techniques to integrate scRNA-seq and seqFISH data in the mouse visual cortex. While single-cells are considered the smallest units and building blocks of each tissue, they still require proper spatial and structural three-dimensional organization in order to assemble into a functional tissue that can exert its physiological function. Single-cell RNA-seq (scRNA-seq) has played a key role in capturing single-cell gene expression profiles, allowing us to map different cell types and states in whole organisms. Despite this remarkable achievement, this technology is based on cellular dissociation and hence does not maintain spatial relationships between single-cells. Emerging technologies now profile the transcriptome of single-cells within their original environment, offering the possibility to examine how gene expression is influenced by cell-to-cell interactions and how it is spatially organized. One such approach is sequential single-molecule fluorescence in situ hybridization (seqFISH [[1](#_Single-cell_in_situ)]), which can identify single molecules at (sub)cellular resolution with high sensitivity.

In contrast with scRNA-seq, seqFISH and many other spatial transcriptomic technologies often pose significant technological challenges, resulting in a small number of profiled genes per cell (10-100s). The newer generation of seqFISH technology (called seqFISH+ [[2](#_Eng_CHL,_Lawson)]) has dramatically enhanced its capacity to profile up to 10,000 genes, but this technology is more complex and costly than seqFISH.

New computational approaches are needed to integrate scRNA-seq and seqFISH data effectively. This hackathon provided seqFISH and scRNA-seq data corresponding to the mouse visual cortex ([[3](#_Tasic_B,_Menon), [4](#_Zhu_Q,_Shah,)]). Our analyses primarily aimed to accurately identify cell types in the seqFISH dataset.

### Data description and access

The scRNA-seq data included transcriptional profiles at a high molecular resolution whereas the seqFISH data provided spatial characterization at a lower molecular resolution. Data were pre-processed for the hackathon and consisted in the measurements of 1,809 genes from 1,723 cells in the scRNA-seq dataset, and 113 genes from 1,597 cells in the seqFISH dataset. There were 113 matching genes between the two data sets that were considered for training and prediction of cell types. For each cell in seqFISH, their XY point coordinates were available. Cell type labels were derived from scRNA-seq analysis [[3](#_Tasic_B,_Menon)] and previous seqFISH/scRNA-seq integration [[4](#_Zhu_Q,_Shah,)] were also provided as reference. Access to easy data and more details can be found at this [link](https://github.com/BIRSBiointegration/Hackathon/tree/master/seqFISH).

###
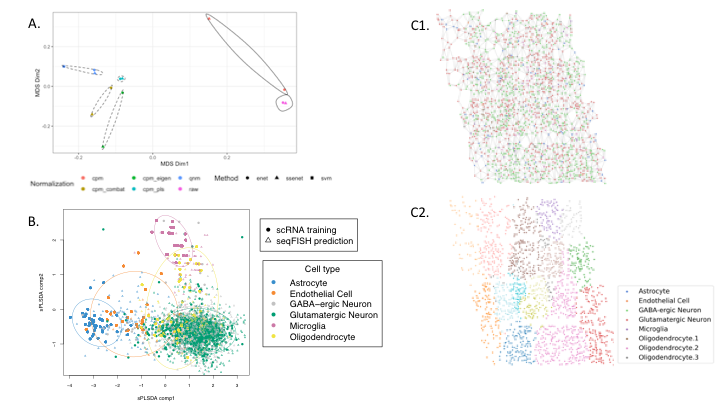
**Figure S1. Overview of seqFISH and scRNA-seq integration analysis**. **A. Challenge 1:** training on the combined datasets. Assessment of cell type prediction using different data normalizations, supervised and semi-supervised classifiers*. Gower distance between each method-normalization pair is depicted on a multidimensional scaling plot. The first dimension (x-axis) separates methods that normalize the scRNA-seq and seqFISH data together (dashed) and separately (solid), showing that normalization had a stronger impact on cell type predictions than the classification method used. **B.** **Challenge 1:** gene selection and cell type prediction in seqFISH. Each classifier was trained using the scRNA-seq data and the cell type labels provided by [[3](#_Tasic_B,_Menon)] to predict cell type labels in the seqFISH data. sPLS-DA was trained on the scRNA-seq data (91% accuracy, 29 genes selected). Cells are represented in the space spanned by the first two components, with 95% confidence ellipse plots. Cells from seqFISH and their predicted cell types were then overlaid and colored according to their cell types according to their predicted component coordinates (71% overall accuracy compared to original study [[4](#_Identification_of_spatially)]; cells predicted as Astrocytes with 90% accuracy). **C.** **Challenge 2:** construction of a spatial network from cells’ positions using Voronoi tessellation after cell type prediction from the scRNA-seq data with SVM. C1: Spatial network of predicted phenotypes C2: Spatial map of areas detected with the neighbors aggregation statistics method. The aggregation statistics can be leveraged to measure the level of cellular heterogeneity within areas, and they can be compared between areas to assess how spatial factors modulate gene expression.

*Normalizations techniques included: one (raw), counts per million (cpm), ComBat batch correction applied to cpm (cpm_combat), scRNA-seq and seqFISH scaled using the first eigenvalue (cpm_eigen), latent variables retained for both datasets after applying Partial Least Squares regression to cpm_eigen normalized data (cpm_pls). Classifiers approaches included a supervised multinomial classifier with elastic net penalty (enet), a semi-supervised multinomial classifier with elastic net penalty (ssenet) and Support Vector Machine (SVM, supervised).

### **Computational challenges**

Two key computational challenges were identified to enable high-resolution spatial molecular resolution. First, we explored several strategies to identify the most likely cell types in the seqFISH dataset based on information obtained from the scRNA-seq (training) dataset. Second, we sought to transfer spatial information obtained from the seqFISH dataset to that of the scRNA-seq dataset.

### Challenge 1: Overlay of scRNA-seq onto seqFISH for resolution enhancement

The mouse visual cortex consists of multiple complex cell types. However, the seqFISH dataset was limited to 113 profiled genes, which were not prioritized based on their ability to discriminate between cell types. Assigning the correct cell identity presents an important challenge. In contrast, the scRNA-seq dataset is transcriptome-wide. This challenge proposed to train models using either all 113 genes or a selection amongst those from the scRNA-seq dataset to then predict cell types from the seqFISH dataset.

Preliminary analyses highlighted that normalization strategies had a significant impact on the final results ([**Figure S1A**](#_28736c3pnd5d)). In addition, although unique molecular identifier (UMI) based scRNA-seq and seqFISH can both be considered as count data, we observed dataset specific biases that could be attributed to either platform (imaging vs. sequencing batch effects) or sample specific sources of variation. We opted to apply a quantile normalization approach that forces a similar expression distribution for each shared gene.

We applied various machine learning and data integration models (see [Vignettes](https://docs.google.com/document/d/12QfLwLdqp4cepMHswlsifiIWRwCSu5UFfjujEWwGGpE/edit#heading=h.p8efz0aoc92s)) and different classification accuracy metrics were considered, such as balanced classification accuracy, over and undersampling techniques to alleviate the major class imbalance in each dataset (90% of cells were excitatory or inhibitory neurons). Two types of classification approaches were considered, supervised and semi-supervised, using a variety of linear and non-linear classifiers such as generalized linear model regularized with elastic net penalty (enet and ssenet), support vector machines (SVM) and sparse Partial Least Squares Discriminant Analysis (sPLS-DA, [5](#_Sparse_PLS_Discriminant)). The supervised approach sPLS-DA fits linear latent components to discriminate known cell types (from scRNA-seq) while allowing for internal gene selection. The balanced classification accuracy of 91% (estimated using 50 x 5-fold cross-validation on the training set) was achieved with a selection of 29 genes ([**Figure S1B**](#_28736c3pnd5d)**)**. If we considered the original study of [[4](#_Identification_of_spatially)] as “ground truth”, or at least to assess potential consensus on prediction, the overall accuracy of the seqFISH cell type predictions was around 71%, with cells from some specific classes correctly predicted with an accuracy greater than 80% (Astrocytes 83%, Microglia 91%). Regarding the list of genes selected, several were found to be associated with specific cell types: Gja1 was reported to regulate astrocytic migration and proliferation [[6](#_Homkajorn_B,_Sims)] and was identified as a marker gene of astrocytes in the original study of [[3](#_Tasic_B,_Menon)] ; Cldn5 encodes a protein that forms tight junction strands, as a physical barrier to prevent solutes and water from passing through endothelial cell sheets [[7](#_RefSeq,_May_2018)]; Omg (oligodendrocyte myelin glycoprotein) is present in Oligodendrocytes; Laptm5 was chosen in [[8](#_The_Transcriptional_Landscape)] to illustrate the expression profiles for the microglial gene module and Tbr1 was found to be a common genetic determinant for the differentiation of early-born glutamatergic neocortical neurons [[9](#_Tbr1_regulates_differentiation)].

Other gene selection procedures were investigated, for example using recursive feature elimination with various combinations of kernels and hyperparameters in SVM. The accuracy was lower than sPLS-DA, but also yielded smaller gene selection sizes than originally proposed by [[4](#_Identification_of_spatially)] ([**Table 1.5**](https://docs.google.com/document/d/12QfLwLdqp4cepMHswlsifiIWRwCSu5UFfjujEWwGGpE/edit#bookmark=id.kt59l9y8gw94)).

While supervised approaches trained on scRNA-seq can then be used to predict cell type in the seqFISH data, other approaches combined both datasets. The semi-supervised ssenet approach builds a model iteratively by combining with cells from both datasets with highest confidence labels before adding more cell type labels until all cells are classified ([**Figure S1A**](#_28736c3pnd5d)). This type of self-training approach might be promising to generalize information to other datasets. The LIGER approach based on integrative non-negative matrix factorization (NMF) integrates both datasets in a subspace based on shared factors, enabling the transfer of cell type labels using a nearest neighbor approach ([**Figure S1D**](#_28736c3pnd5d)). However, the clustering accuracy metrics showed that such an alignment strategy proved challenging in this study.

These analyses highlighted typical challenges when conducting statistical learning from similar data types (gene expression) measured using different assays. Normalisation and pre-processing of data required to address (batch) variation between assays to enable accurate cell type prediction. In addition, in order to predict cell types from one assay to the other, we needed to rely on an established ‘ground truth’ for training the classifier model. A semi-supervised approach could allow refinement of cell types predictions for uncertain classes in seqFISH, however, such an analysis requires to either correct or take into account batch effects between assays. Our attempts using this approach did not seem to improve the quality of prediction (see [Table 1.4](https://docs.google.com/document/d/12QfLwLdqp4cepMHswlsifiIWRwCSu5UFfjujEWwGGpE/edit#bookmark=id.nfsy56bc6gm2) and [Table 1.6](https://docs.google.com/document/d/12QfLwLdqp4cepMHswlsifiIWRwCSu5UFfjujEWwGGpE/edit#bookmark=id.rwiepu7otc9u)). The evaluation of the quality of the prediction in the seqFISH was limited by the lack of biological knowledge, the non-targeted nature of the genes sequenced - which are not necessarily characteristics of those cell types, and the exploratory nature of our analyses. Finally, the type of classifier (linear or non-linear, supervised or semi-supervised) and gene selection strategies were found to influence the performance of the methods, whose evaluation required an adequate choice of metrics to account for cell type imbalance.

### Challenge 2: Identifying spatial expression patterns at the tissue level through the integration of gene expression and spatial cellular coordinates

While most tools originally developed for scRNA-seq data can be adapted for spatial transcriptomic datasets, methods to extract sources of variation from spatial factors are still lacking. Novel methods that can integrate the information obtained from gene expression with that of the spatial coordinates from each cell or transcript (for sub-cellular resolution) within a tissue of interest are needed.

To identify spatial expression patterns in the seqFISH dataset, some analyses focused on forming a spatial network based on Voronoi tessellation [[10](#_Coullomb_A,_Pancaldi), [11](#_Dries_R,_Zhu)]. The gene expression of each cell was spatially smoothed by calculating the average gene expression of all neighboring cells. UMAP was applied to the smoothed and aggregated data matrix to identify cell clusters with a density-based clustering approach ([**Figure S1D**](#_28736c3pnd5d)). Interestingly, these results showed that the obtained clusters themselves are spatially separated and do not necessarily overlap with specific cell types, suggesting that the spatial dimension cannot be captured from the expression data only.

An unanswered question is whether the identified combinatorial spatial patterns can be extracted directly from scRNA-seq data, as previous studies have shown cellular mapping between gene expression profiles and known spatial locations [[12](#_Spatial_reconstruction_of), [13](#_Single-Cell_Multi-omic_Integration)]. However, this still constitutes both a technological and analytical challenge that will require careful benchmarking in the near future.

Combining spatial information with other high dimensional omics data will allow for a more comprehensive view to discover fundamental biological processes regarding the regulation of gene expression by cells environment and interactions, the emergence of spatial patterns, and how these patterns play a role for developmental processes and diseases such as solid tumour growth. However, many challenges still remain unanswered, such as integration of omics with spatial data, appropriate computational resources and biological interpretation of the results that are obtained.

## **S2. Case study for cross-study and cross-platform analysis: spatial proteomics**

### **Overview and biological question**

Whereas the previous hackathon included samples from the same biological conditions, this hackathon challenged participants to analyze two datasets obtained from different single-cell targeted proteomics (antibody-based) technologies, applied to breast cancer tissue of different patient cohorts, from different laboratories. Both studies examined the tumor-immune microenvironment in primary breast cancer: Wagner, et al. used Mass Cytometry (CyTOF) to assay 73 proteins across two panels (tumor and immune) in 194 tissue samples from 143 subjects, of which 6 patients had triple-negative negative breast cancer [[14](#_A_Single-Cell_Atlas)], while Keren, et al. applied Multiplexed Ion Beam Imaging (MIBI) to quantify spatial in-situ expression of 36 proteins in 41 triple-negative breast cancer patients [[15](#_A_Structured_Tumor-Immune)] ([**Figure S2A**](#_16scdbg66noe)). Easy access data and further details are available at this [link](https://github.com/BIRSBiointegration/Hackathon/tree/master/sc-targeted-proteomics).

This hackathon focused on an integrative data analysis across studies and platforms given a limited overlap in features. Three main challenges emerged. The first challenge investigated whether analytical methods could integrate partially-overlapping proteomic data collected on different patients with similar phenotypes, and whether measurements from one technology (MIBI spatial location and expression of proteins) could be transferred and used to predict information in the second technology (e.g., spatial expression patterns of proteins measured on CyTOF). The second challenge explored the added value of spatial technologies to uncover additional information about immune cell populations in breast cancer beyond cell composition. The third challenge examined the possibility to integrate data from patients with heterogeneous phenotypes given few common features and no overlap in biological samples.

###
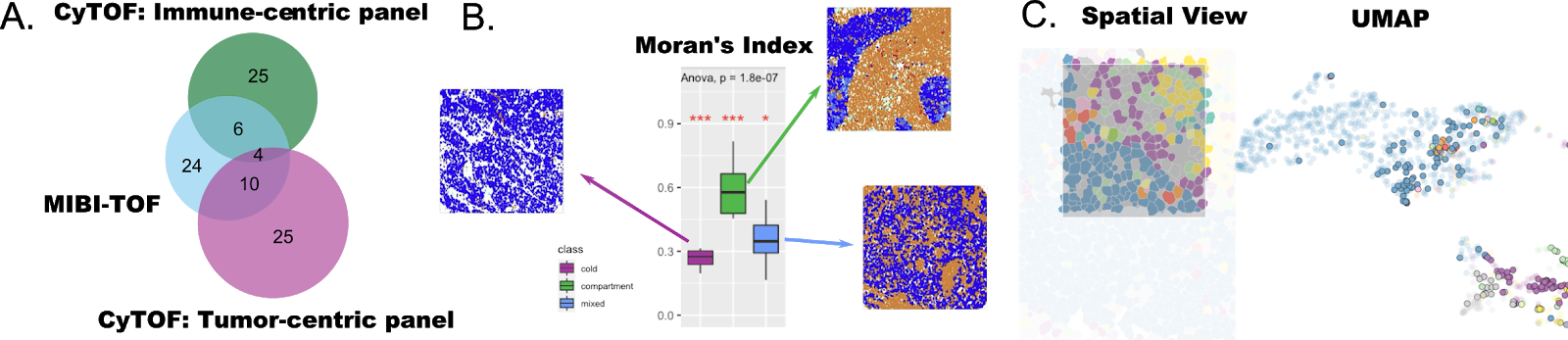
**Figure S2. A** The datasets selected for this hackathon had limited overlap in features (MIBI-TOF, CyTOF immune-centric panel, and CyTOF tumor-centric panel; illustrating *Challenge 1*) **B** Spatial analysis with Moran’s index computed on Gabriel graph shown in boxplot according to tumor/immune status showing a significant difference between groups (Red asterisks indicate significance of an ANOVA of each group with all others with p-value from an overall ANOVA across the three groups reported; exploring *Challenge 2*). **C** Cells can be studied through either spatial or gene expression relationships, and correspondences between both representations can be highlighted through linked brushing, an interactive visualization technique (exploring *Challenge 2*).

### **Computational challenges**

### Challenge 1: Limited overlap between protein features across studies

There were only 20 proteins that were assayed in both the CyTOF [[14](#_A_Single-Cell_Atlas)] and MIBI-TOF [[15](#_A_Structured_Tumor-Immune)] studies ([**Figure S2A**](#_16scdbg66noe)), which precluded integration of features at the level of gene set or pathways and required the use of surrogate measures for cross-study association. The majority of proteins were cell-type markers or biomarkers targets of breast cancer therapeutic intervention, providing the opportunity to perform cross-study integration of cell type proportions in tumor tissue samples.

Several semi-supervised and supervised algorithms were applied to transfer cell labels and cell compositions from one dataset to the second (see [Table 1](https://docs.google.com/document/d/12QfLwLdqp4cepMHswlsifiIWRwCSu5UFfjujEWwGGpE/edit#heading=h.p8efz0aoc92s)). Random forest was considered to capture the hierarchical structure of cell lineage and perform feature transfer learning of cell type labels, using an adaptation of the prediction strength approach [[16](#_Cluster_Validation_by)] to assess model robustness: first, a model was trained on the labeled dataset, then used to predict labels in the unlabeled dataset; next, a second model was trained based on the second dataset with the newly predicted labels; finally, the ability of the second model to recover the correct original labels when making predictions on the labeled dataset was assessed. Mapping cells from CyTOF to imaging with spatial information was handled by solving an entropic regularization optimal transport problem [[17](#_Optimal-Transport_Analysis_of), [18](#_Inferring_spatial_and)], using the cosine distance of the common proteins between the two datasets as transport cost. The constructed optimal transport plan can be considered as likelihood of cells from one modality mapped to cells from the other modality, which allows the prediction of protein expression measured only in CyTOF on imaging data. After cluster analysis of the resulting imputed expression matrix, sub tumour cell type could be identified that was not revealed in the original matrix.

Another issue encountered with this challenge was that the different scales of protein expression across technologies meant that cell compositions could not be integrated using correlation of the expression of protein markers, as some cell markers were expected on a range of cell types (e.g. CD45), while others were more specialized and appeared in only a subset of those cells (e.g. CD4). Other challenges associated with cell composition analysis of proteomics data included uncertainty about antibody specificity and consistency between studies; specific sensitivity and specificity of protein markers for cell types and tissues; and disease heterogeneity. Cell type assignment was also a significant challenge, as it relied on manually curated protein annotation, and was therefore dependent on domain-specific knowledge (e.g. CD4 is expressed by T-cells). To date, methods for cell type assignment, classification or extraction of differentially expressed proteins cannot easily be applied to targeted proteomics. There is thus an urgent need for a unifying map between cells present in different datasets, and for annotation resources to provide quality metric or priors of protein cell type markers. The construction of protein expression atlases would support cell type classification, even if antibodies used and their performances might vary between laboratories.

### Challenge 2: spatial analysis of protein expression

CyTOF mass spectrometry data provided protein expression and counts/composition of cells in breast tumor-immune environment, while the MIBI-TOF data provided spatial information that quantified cell attributes (shape, size, spatial coordinates) in addition to expression levels. These two data sets thus provide the opportunity to examine protein expression, cell microenvironment, and predict cell-cell interactions and the cellular community ecosystem.

Spatial information can be encoded as a set of XY coordinates (cell centroid), a line (e.g. tumor-immune boundary), or a polygon, which is a closed plane defined by a number of lines and can define complex shapes such as a cell or a community of cells. Spatial protein expression can be summarized using spatial descriptive statistics, such as the autocorrelation of the expression of a protein within a neighborhood of polygons, using techniques developed in geographical information science or ecology to assess whether a spatially measured variable has a random, dispersed or clustered pattern [[19](#_Lundberg_E,_Borner)].

We investigated whether expression data could be used to predict spatial properties of tissue samples using a variety of approaches (see [Table 1](https://docs.google.com/document/d/12QfLwLdqp4cepMHswlsifiIWRwCSu5UFfjujEWwGGpE/edit#heading=h.p8efz0aoc92s)). A K-nearest neighbor graph was used to build spatial response variables and random forest model trained from expression data to predict spatial features. A topic model was trained on protein expression of CyTOF and MIBI-TOF data to predict cell co-locations of CyTOF immune cells where 10% of MIBI-TOF considered test data. Among the five topics identified, the first topic was dominated in most of the immune cells from CyTOF data and the other four dominated in all other cells. Prognostic performance of different higher level spatial metrics was also examined using Moran’s Index with a sphere distance, cell type localisation using nearest neighbour correlation, or cell type interaction composition with Ripley’s L-function. Cox models with fused lasso penalty and random forest survival models were then fitted based on clinical features such as tumor stage, tumor grade, age and tumor size, as well as cell type composition. The spatial metrics were found to be predictive, especially in triple negative breast cancer where clinical features such as grade are often poor prognostics. Further investigation of Moran’s Index using a graph-based neighborhood measure (Gabriel graph, based on Delaunay triangulation; as opposed to sphere distance) found the values of this metric differed significantly between the three prognostic tumor scores described by [[15](#_A_Structured_Tumor-Immune)] ([**Figure S2B**](#_16scdbg66noe)). This challenge demonstrated the prognostic potential of spatial single-cell proteomics data and underscores the need to develop new spatial measures specifically for these data.

### Challenge 3: Fourth corner integration of data at the level of phenotype

Cross-study integration also raises the challenge of non-overlapping biological samples but with similar phenotypes. In this challenge, the aim was to identify biomarkers from the different data types to predict phenotype, and, more importantly, to explore concordance among markers selected across multiple studies and datasets. Depending upon how well these markers can be transferred across datasets, as well as the amount of distinctive information encoded by different markers, integrating datasets with only some overlap in markers could potentially provide more biological insight than from individual ‘omics studies. To consider this third challenge, phenotypic data (such as the cell attributes) were the critical factors that should be used to link the two datasets ([**Figure 2D**](https://docs.google.com/document/d/12QfLwLdqp4cepMHswlsifiIWRwCSu5UFfjujEWwGGpE/edit#heading=h.g4iaw3kox5pd)).

Integrating patient phenotype measures such as grade, stage and overall survival is one first step that we were able to achieve. However, integrating proteins from data sets that used different approaches to cell type annotation and had limited proteins in common was extremely challenging. Borrowing from ecology and the French school of ordination, this problem can be described as a case of the fourth corner problem (or RLQ, [**Figure 2D**](https://docs.google.com/document/d/12QfLwLdqp4cepMHswlsifiIWRwCSu5UFfjujEWwGGpE/edit#heading=h.g4iaw3kox5pd)). Briefly, given two ’omics data where both features and samples are non overlapping, and phenotypical data are available for each omics data, multiplying the two phenotypical factors should derive a bridging matrix that links the features of two omics data. This requires the two phenotypical matrices to be multiplicable, i.e. describing the same phenotypical factors. The fourth corner RLQ can be solved using matrix decomposition [[20](#_Matching_species_traits), [21](#_Linking_trait_variation)]. However, this approach was not attempted in this hackathon.

## **S3. Case study for epigenetic regulation: scNMT-seq**

### **Overview and biological question**

scRNA-seq technologies have enabled the identification of transcriptional profiles associated with lineage diversification and cell fate commitment [[22](#_Using_single‐cell_genomics)], but the role of epigenetic layers still remains poorly understood [[23](#_Reprogramming_the_Methylome:)]. In contrast to the previous two hackathons, which leveraged datasets from complementary technologies to enable high molecular and spatial resolution of biological systems, this hackathon used datasets spanning disparate molecular scales (e.g. DNA and RNA measurements) to improve our understanding of cell fate decisions using scNMT-seq.

scNMT-seq is one of the first experimental protocols that enable simultaneous quantification of RNA expression and epigenetic information from individual cells [[24](#_scNMT-seq_enables_joint)]. Briefly, cells are incubated with a GpC methyltransferase enzyme that labels accessible GpC sites via DNA methylation. Thus, GpC methylation marks can be interpreted as direct read-outs for chromatin accessibility, whereas CpG methylation marks can be interpreted as endogenous DNA methylation. By physically separating the genomic DNA from the mRNA, scNMT-seq can profile RNA expression, DNA methylation and chromatin accessibility read-outs from the same cell. This third hackathon focused on data integration strategies to detect global covariation between RNA expression and DNA methylation variation from scNMT-seq data in a mouse gastrulation study [[25](#_Argelaguet_R,_Clark)].

Gastrulation is a major lineage specification event in mammalian embryos that is accompanied by profound transcriptional rewiring and epigenetic remodeling [[26](#_Hanna_CW,_Demond)]. In this study, four developmental stages were profiled, spanning exit from pluripotency to germ layer commitment (E4.5 to E7.5).

### Data description and access

For simplicity in our analyses, we focused on the integration of RNA expression and DNA methylation. The latter were quantified over the following genomic contexts: gene bodies, promoters, CpG islands, and DHS open sites. The number of features ranged from 6,673 (DHS open sites) to 18,345 (gene expression), with a percentage of missing values from 18% (gene bodies) to 59% (DHS open sites), see [**Figure S3A**.](#_8wnd3rjmg9vt) A total of 799 cells passed quality control. Preliminary analyses using dimensionality reduction methods confirmed that all four embryonic stages could be separated on the basis of RNA expression ([**Figure S3B**](#_8wnd3rjmg9vt)). Easy access data and further details are available at this [link](https://github.com/BIRSBiointegration/Hackathon/tree/master/scNMT-seq).

### Computational challenges

The main challenge was to leverage the multi-faceted nature of measurements to better resolve the single-cell subpopulations from distinct embryonic stages.

We considered 3 computational strategies (see [Table 1](https://docs.google.com/document/d/12QfLwLdqp4cepMHswlsifiIWRwCSu5UFfjujEWwGGpE/edit#heading=h.p8efz0aoc92s)): MOSAIC (Multi-Omics Supervised Integrative Clustering algorithm inspired by survClust [[27](#_Pan-cancer_identification_of)]) classifies samples by creating weighted distance matrices across data modalities, where the weights are defined as the maximum of the ratio of cluster specifc vs. population log likelihoods ([**Figure S3C**](#_8wnd3rjmg9vt)). LIGER is an unsupervised non-negative matrix factorization model for manifold alignment that assumes a common feature space by aggregating DNA methylation over gene-centric elements (promoters or gene bodies) but allows cells to vary between data modalities [[13](#_Single-Cell_Multi-omic_Integration)] ([**Figure S3D**](#_8wnd3rjmg9vt)). Multi-block sparse Projection to Latent Structures (multiblock sPLS), is a sparse generalization of canonical correlation analysis that maximizes paired covariances between the RNA data set and each of the other genomic context data sets [[28](#_Variable_selection_for), [29](#_mixOmics:_An_R)] ([**Figure S3E**](#_8wnd3rjmg9vt)).


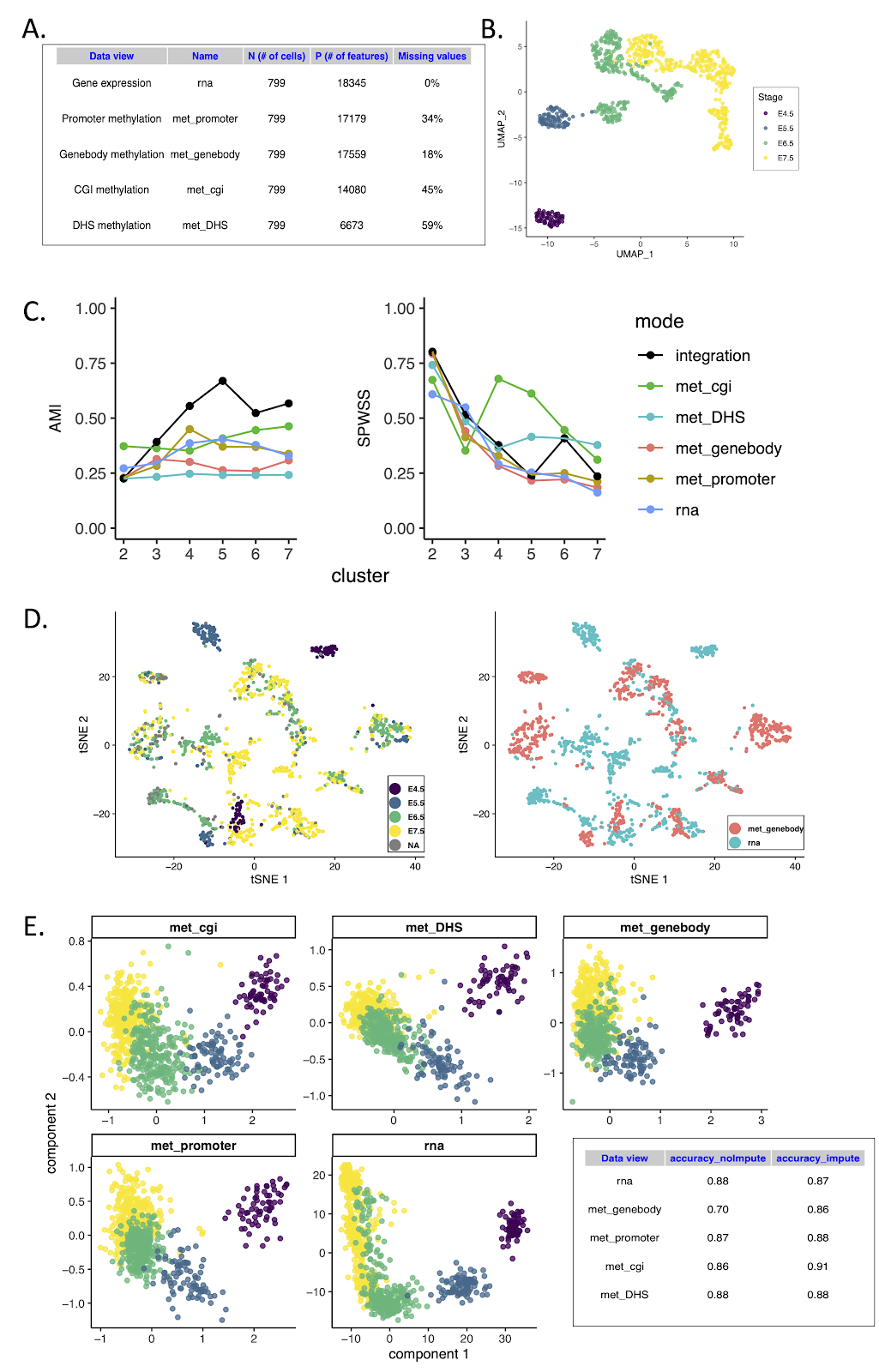


### **Figure S3. Overview of hackathon analyses for the scNMT-seq challenge.** **A** Summary of the data modalities analyzed, including different putative regulatory regions. **B** UMAP of RNA measurements using 671 highly variable genes shows separation of the four embryonic stages. **C** Supervised analysis using view-specific and integrative distance measures with MOSAIC: The integration identified five clusters of cell populations based on Adjusted Mutual Information and Standardized Pooled Within Sum of Squares that outperforms individual (single omics) analyses. **D** LIGER joint alignment using gene body methylation and RNA expression: cells are colored by stage (left) or original data modality (right). **E** Unsupervised integration using multi-block sPLS: cells are projected into the space spanned by each data view components that are maximally correlated. For performance assessment, two types of analyses were considered, either by omitting the missing DNA methylation values or incorporating imputed values. K-means clustering analysis based on the multi-block sPLS components was used to calculate balanced accuracy measures.

### Challenge 1: defining genomic features and data integration across genomic contexts

The first challenge presented in this hackathon was how to define input data. The output of single-cell bisulfite sequencing are binary DNA methylation measurements for individual CpG sites. Integrative analysis at the CpG level is extremely challenging due to the sparsity levels, the binary nature of the read-outs, and the intricacy in interpretation of individual dinucleotides. To address these problems, DNA methylation measurements are typically aggregated over pre-defined sets of genomic elements (i.e. promoters, enhancers, etc.). This pre-processing step reduces sparsity, permits the calculation of binomial rates that are approximately continuous and can also improve interpretability of the model output.

We observed remarkable differences between genomic contexts on the integration performance. In MOSAIC, stages were better separated when using DNA methylation measurements on promoter regions and at least four clusters (AMI=0.45). Interestingly, this setting performed better than using RNA expression alone (AMI=0.40). Notably, when using an integrated solution across data modalities, stages were better classified (AMI = 0.68) ([**Figure S3C**](#_8wnd3rjmg9vt)). LIGER, which was also applied in spatial transcriptomics hackathon, requires a common feature space to perform alignment of cells when profiled for different data modalities. Since this hackathon provides unambiguous cell matching between the data modalities, it represents a gold standard for testing this approach. LIGER was applied to gene expression and gene body methylation: the poor alignment suggested a complex coupling of gene expression and gene body methylation during gastrulation ([**Figure S3D**](#_8wnd3rjmg9vt)). Finally, multi-block sPLS identified co-varying components between RNA expression and DNA methylation that separated cell stages in all putative regulatory contexts considered ([**Figure S3E**](#_8wnd3rjmg9vt)). Taken altogether, these results confirmed that the appropriate selection of the feature space (data set) is critical for a successful integration with RNA expression.

### Challenge 2: Missing values in DNA methylation

Single-cell bisulfite sequencing protocols are limited by incomplete CpG coverage because of the low amounts of starting material. Nonetheless, in contrast to scRNA-seq, missing data can be distinguished from dropouts. Integrative methods can be divided into approaches that can handle missing values (e.g. MOSAIC, multi-block sPLS which omit missing values during inference), or approaches that require *a priori* imputation (e.g. LIGER).

We compared the integration performance of multiblock sPLS either with original or with imputed data. The missing values were inferred using nearest neighbor averaging (as implemented in the impute package [[30](#_impute)]) in the methylation data. The components associated with each data set showed varying degrees of separation of the embryonic stages, depending on the genomic contexts ([**Figure S3E**](#_8wnd3rjmg9vt)). Accuracy measures based on k-means clustering analysis on the multiblock sPLS components showed that gene body methylation components were better at characterizing embryonic stage after imputation (from 70% with original data to 86% after imputation).

Missing values in regulatory context data represent a topical challenge in data analysis, and further methodological developments are needed to either handle and accurately estimate missing values.

### Challenge 3: Linking epigenetic features to gene expression

One of the main advantages of scNMT-seq is the ability to unbiasedly link epigenetic variation with gene expressio n [[24](#_scNMT-seq_enables_joint)]. Transcriptional activation is associated with specific chromatin states near the gene of interest. This includes deposition of activatory histone marks such as H3K27ac, H3K4me3 and H3K36me3, binding of transcription factors, promoter and/or enhancer demethylation and chromatin remodeling [[31](#_The_interplay_of)]. All these events are closely interconnected and leave a footprint across multiple molecular layers that can be (partially) recovered by performing an association analysis between a specific chromatin read-out and mRNA expression [[32](#_Single-Cell_Multi-omics:_An)]. However, given the large amount of genes and regulatory regions, this task can become prohibitively large, with the associated multiple testing burden. In addition, some of our analyses have shown that the correlations between epigenetic layers and RNA expression calculated from individual genomic features can be generally weak or spurious.

A practical and straightforward approach from a computational perspective involves considering only putative regulatory elements within each gene’s genomic neighborhood. Nonetheless, this might miss important links with regulatory elements located far away from the neighborhood.

In recent years, chromosome conformation capture experiments have uncovered a complex network of chromatin interactions inside the nucleus connecting regions separated by multiple megabases along the genome and potentially involved in gene regulation [[33](#_Methods_for_mapping), [34](#_Long-range_enhancer–promoter_contac)]. Early genome-wide contact maps generated by HiC uncovered domains spanning on the order of 1 Mb (in humans [[35](#_Topological_Domains_in)]) within which genes are likely to be coordinately regulated [[36](#_A_3D_Map), [37](#_The_role_of)], in ways that are not entirely understood [[38](#_Independence_of_chromatin)]. Thus, a second strategy to associate putative regulatory elements and genes is to build on existing promoter-centered chromatin contact networks [[39](#_The_pluripotent_regulatory), [40](#_Using_GARDEN-NET_and)] to restrict the association analysis to putative regulatory elements that are in 3D contact with genes [[41](#_Lineage-Specific_Genome_Architectur), [42](#_A_compendium_of), [43](#_Widespread_reorganisation_of)]. Although this is a promising strategy to reduce the complexity of the association analysis, most of our 3D interaction datasets are produced in bulk samples and it is so far unclear how much of these structures are preserved across individual cells [[44](#_Szabo_Q,_Jost)]. While single-cell conformation capture experiments remain limited by data sparsity and high levels of technical noise [[45](#_Zhou_T,_Zhang)], we envision that technological advances in this area will deepen our understanding of the regulatory roles of chromatin states.

## S4. Further considerations on benchmarking

Another strategy for benchmarking is to use cross-validation within a study, or conduct cross-study validation to assess whether solutions found by multi-modal methods generalize to held-out observations or held-out studies. The latter was attempted in hackathon 2 but where ground truth was unknown.

#### Cross-validation within study

Cross-validation within a representative multi-modal study is one possible approach for quantitative assessment for unbiased comparison of methods. We note that the approach of cross-validation – in which observations are split into folds or left out individually for assessing model fit – has been used often for parameter tuning within methods, or for other aspects of model selection [[29](#_mixOmics:_An_R), [46](#_DIABLO:_an_integrative)-[56](#_Identifying_multi-layer_gene)].

Similarly, permutation has been used to create null datasets, either as a demonstration that a particular method is not overfitting, or for parameter tuning, where the optimal parameter setting should result in a model score that is far from the null distribution of model scores [[57](#_Cross-platform_comparison_and), [58](#_Extensions_of_Sparse), [59](#_MOGSA:_Integrative_Single)]. Cross-validation is particularly useful as a quantitative assessment of a method’s self-consistency, even though it cannot determine the *accuracy* of a method in a completely unbiased way if we do not have access to an external test data set for further confirmation.

As part of hackathon 3, we performed a cross-validation analysis of the scNMT-seq dataset using MOFA+ ([Table 1.12](https://docs.google.com/document/d/12QfLwLdqp4cepMHswlsifiIWRwCSu5UFfjujEWwGGpE/edit#bookmark=id.8owin94f7epz)). Strong relationships found among pairs of modalities in training data were often reproduced in held out cells ([**Figure S5**](#_h9ndp0w4ryf9)). This analysis also revealed that we could reliably match dimensions of latent space across cross-validation folds. Previous evaluations of multi-modal methods have focused only on the top latent factor [[60](#_Consistency_and_overfitting)], however, we showed in our analyses, many latent factors can be reliably discovered in held out cells in studies of complex biological processes such as the differentiation of embryonic cells.

For clustering assessment, several studies have used resampling or data-splitting strategies to determine prediction strength [[16](#_Cluster_Validation_by), [61](#_Bootstrapping_cluster_analysis:), [62](#_A_prediction-based_resampling), [63](#_A_Three-Gene_Model)]. These techniques could be further extended in a multi-modal setting for clustering of cells into putative cell types or cell states. Community-based benchmarking efforts in the area of multi-modal data analysis could follow the paradigm of the [DREAM Challenges](http://dreamchallenges.org/), with multi-modal training data provided and test samples held out, in order to evaluate the method submissions from participating groups.

#### Cross-validation between studies

Our benchmarking hackathons have emphasized the need to access external studies for methods assessment and validation, where either the ground truth is based on biological knowledge of the system being studied, or via high-quality control experiments where the ground truth (e.g. cell type labels) are known ([**Figure S5A**](#_h9ndp0w4ryf9)). To take advantage of all data and technologies available, cross-study validation could also extend to cross-platform to assess whether relationships discovered in one dataset are present in other datasets, such as looking across single-cell and bulk omics, as was recently proposed in [[64](#_A_simple,_scalable)].

## S5. Further considerations on results interpretation

#### Reasoning by analogy with geospatial problems

Multiple domains of knowledge can be combined easily if there is a common coordinate system, as in geospatial analyses. This is often a goal in multi-modal or conjoint analyses, when the first step is to find a common compromise or consensus on which to project each of the individual modalities. Conjoint analyses also known as STATIS [[65](#_The_ACT_(STATIS)] was a very early multi-modal method designed as “PCA of PCAs” where the first step in the analyses was to identify the commonalities between different modalities and define a consensus onto which the individual data sets were projected [[65](#_The_ACT_(STATIS)]. STATIS can be considered as an extension of the class of matrix decomposition methods to data cubes. Many extensions to matrix decompositions have since been designed for multi-modal data, [[66](#_Multitable_Methods_for)] offer s an overview of the relations between many of them.

The spatial coordinate system analogy can be pursued further by finding a “consensus space” that provides a common coordinate system. Thus, by creating an abstract coordinate space, we can leverage methods developed for true spatial co-occurrences, and evaluate these co-occurrences in abstract spatial coordinates as an effective strategy for creating layered maps despite the the absence of a physical coordinate system. There are however pitfalls in using very sophisticated dimension reduction techniques which lead to over-interpretation or misinterpretation of spatial relations. One such example is the size and closeness of clusters in t-SNE which do not represent true densities or similarities in the original data.

## S6. Further considerations on software

We used the MultiAssayExperiment integrative data class from Bioconductor to enable the collation of standard data formats, easy data access, and processing. It uses the S4 object-oriented structure in R [[67](#_MultiAssayExperiment), [68](#_Ramos_M,_Schiffer)] and includes several features to support multi-platform genomics data analysis, to store features from multiple data modalities (e.g. gene expression units from scRNA-seq and protein units in single-cell proteomics) from either the same or distinct cells, biological specimen of origin, or from multiple dimensions (e.g. spatial coordinates). This class also enables to store sample metadata (e.g. study, center, phenotype, perturbation) and provides a map between the datasets from different assays for downstream analysis. The input data were stored as MultiAssayExperiment objects that were centrally managed and hosted on ExperimentHub [[69](#_ExperimentHub)] as a starting point for all analyses. The SingleCellMultiModal package was used to query the relevant datasets for each analysis [doi:10.18129/B9.bioc.SingleCellMultiModal] ([**Figure S5**](#_h9ndp0w4ryf9)).

##

###
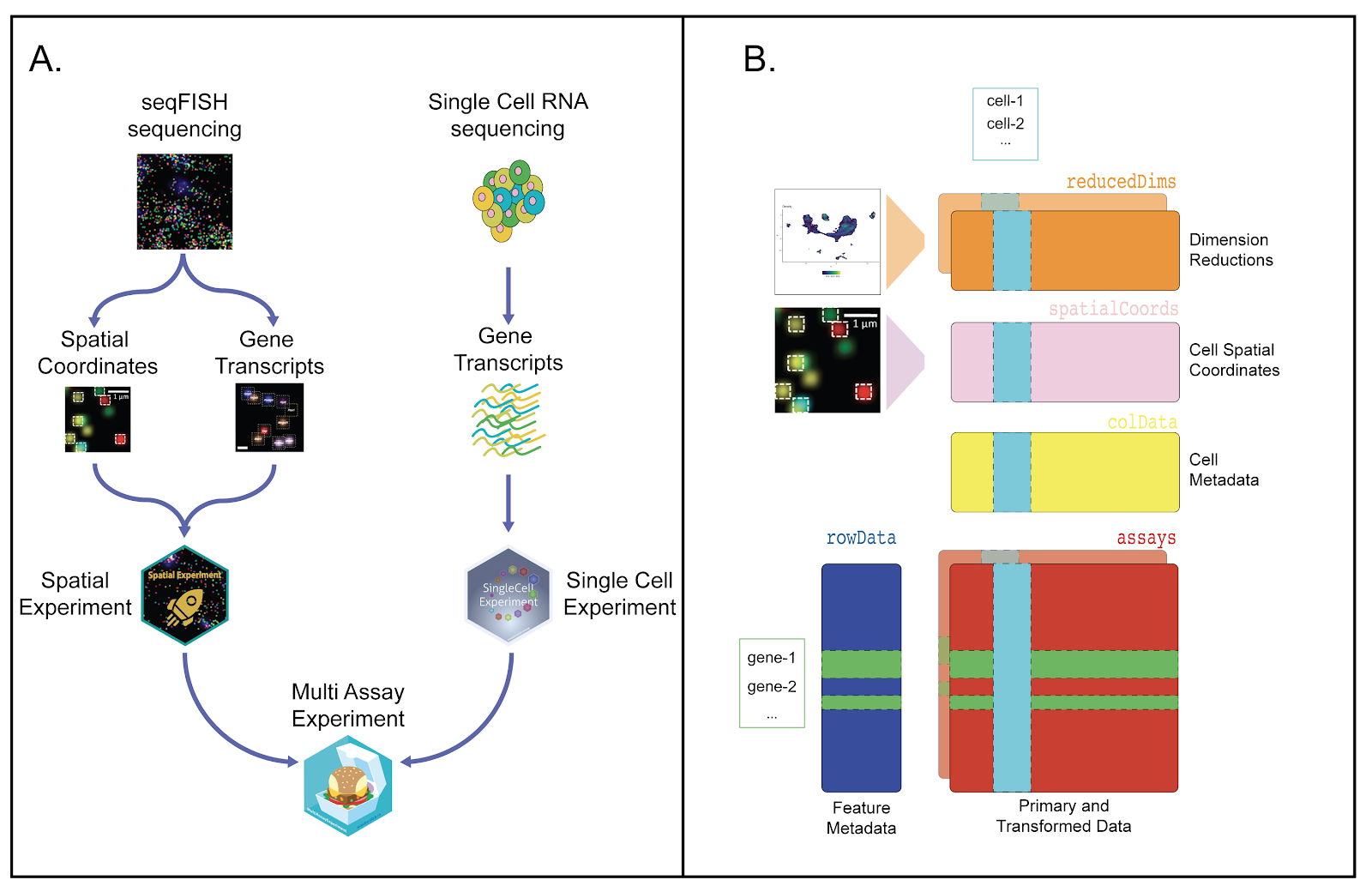
**Figure S4. A** Software infrastructure using Bioconductor for the first hackathon to combine seqFISH-based SpatialExperiment and SingleCellExperiment instances into a MultiAssayExperiment. **B** To combine these two different experiments, the seqFISH data were stored into a SpatialExperiment S4 class object, while the scRNA-seq data were stored into a SingleCellExperiment class object [[70](#_SingleCellExperiment)]. These objects were then stored into a MultiAssayExperiment class object and released with the SingleCellMultiModal Bioconductor package [[71](#_SingleCellMultiModal)].

###
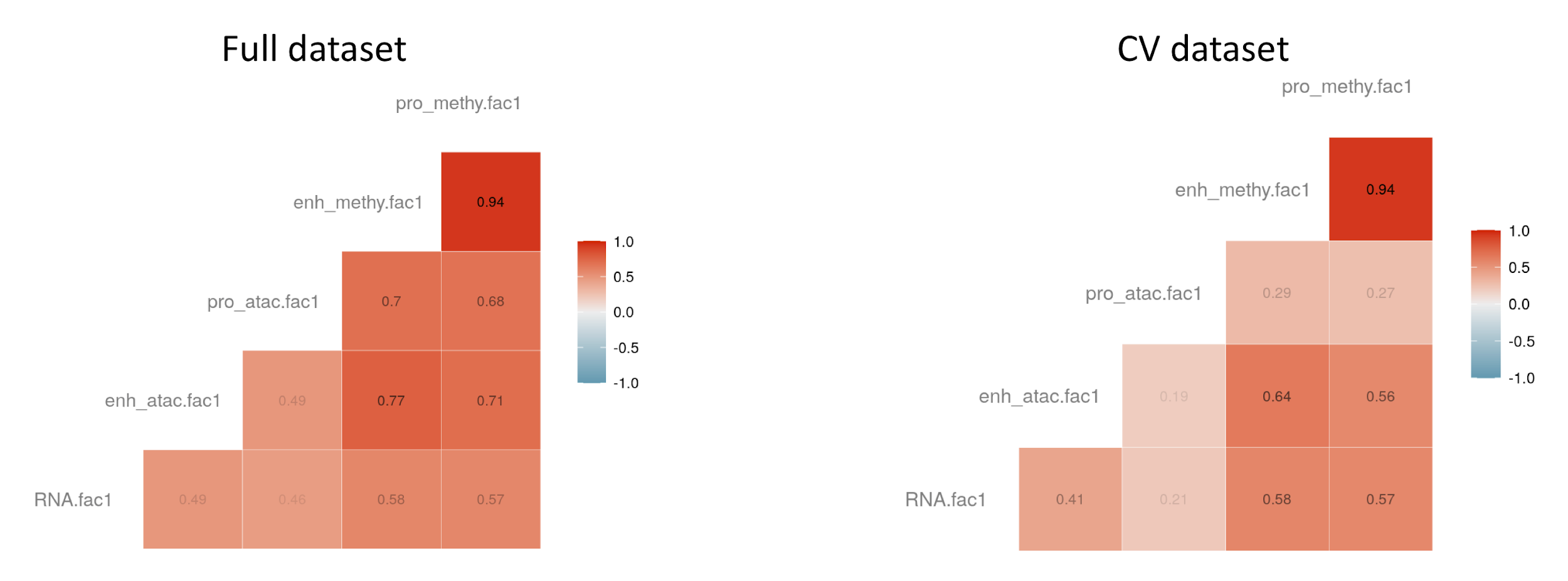
**Figure S5.** scNMT-seq study: correlations with linear projections (MOFA+) evaluated without (top) or with (bottom) cross-validation, showing robust associations between datasets when a subset of cells is held out.

## Online resources

- Online resource 1: Three hackathon easy access datasets: <https://github.com/BIRSBiointegration/Hackathon>
- Online resource 2: R packages with open source reproducible vignettes (12 vignettes): <https://github.com/BIRSBiointegration/Hackathon/blob/master/analysis-vignettes.md>

# **References**

### Lubeck E, Coskun A, Zhiyentayev T et al.: Single-cell in situ RNA profiling by sequential hybridization. Nat Methods. 2014 11, 360–361. <https://doi.org/10.1038/nmeth.2892>

### Eng CHL, Lawson M, Zhu Q et al.: Transcriptome-scale super-resolved imaging in tissues by RNA seqFISH+. Nature 2019 568, 235–239. <https://doi.org/10.1038/s41586-019-1049-y>

### Tasic B, Menon V, Nguyen T et al.: Adult mouse cortical cell taxonomy revealed by single cell transcriptomics. Nat Neurosci. 2016 19, 335–346. <https://doi.org/10.1038/nn.4216>

### Zhu Q, Shah, S, Dries, R et al.: Identification of spatially associated subpopulations by combining scRNAseq and sequential fluorescence in situ hybridization data. Nat. Biotechnol. 2018 36, 1183–1190. <https://doi.org/10.1038/nbt.4260>

### Lê Cao KA, Boitard S, Besse P: Sparse PLS discriminant analysis: biologically relevant feature selection and graphical displays for multiclass problems. BMC Bioinformatics 2011 12, 253. <https://doi.org/10.1186/1471-2105-12-253>

### Homkajorn B, Sims NR, Muyderman H: Connexin 43 regulates astrocytic migration and proliferation in response to injury. Neurosci Lett. 2010 17;486(3):197-201. <https://doi.org/10.1016/j.neulet.2010.09.051>

1. O'Leary, N.A., Wright, M.W., Brister, J.R., Ciufo, S., Haddad, D., McVeigh, R., Rajput, B., Robbertse, B., Smith-White, B., Ako-Adjei, D. and Astashyn, A.: Reference sequence (RefSeq) database at NCBI: current status, taxonomic expansion, and functional annotation. Nucleic acids research 2016, *44*(D1), pp.D733-D745.
2. Bonham LW, Sirkis DW, Yokoyama JS: The Transcriptional Landscape of Microglial Genes in Aging and Neurodegenerative Disease Front. Immunol. 2019. <https://doi.org/10.3389/fimmu.2019.01170>
3. Hevner RF, Shi L, Justice N, Hsueh Y, Sheng M, Smiga S, Bulfone A, Goffinet AM, Campagnoni AT, Rubenstein JLR: Tbr1 regulates differentiation of the preplate and layer 6. Neuron 2001 29(2):353-66. <https://doi.org/10.1016/S0896-6273(01)00211-2>
4. Coullomb A, Pancaldi V: Tysserand—fast and accurate reconstruction of spatial networks from bioimages. Bioinformatics 2021.  <https://doi.org/10.1093/bioinformatics/btab490>
5. Dries R, Zhu Q, Dong R et al.: Giotto: a toolbox for integrative analysis and visualization of spatial expression data. Genome Biol. 2021 22, 78. <https://doi.org/10.1186/s13059-021-02286-2>
6. Satija R, Farrell J, Gennert D. et al.: Spatial reconstruction of single-cell gene expression data. Nat Biotechnol. 2015 33, 495–502. <https://doi.org/10.1038/nbt.3192>
7. Welch JD, Kozareva V, Ferreira A, Vanderburg C, Martin C , Macosko EZ: Single-Cell Multi-omic Integration Compares and Contrasts Features of Brain Cell Identity. Cell 2019. <https://doi.org/10.1016/j.cell.2019.05.006>
8. Wagner J, Rapsomaniki MA, Chevrier A, Anzeneder T, Langwieder C, Dykgers A, Rees M, Ramaswamy A, Muenst S, Soysal SD, Bodenmiller B, et al.: A Single-Cell Atlas of the Tumor and Immune Ecosystem of Human Breast Cancer. Cell 2019. <https://doi.org/gfzbz7>
9. Keren L, Bosse M, Marquez D, West R, Bendall SC, Angelo M, et al.: A Structured Tumor-Immune Microenvironment in Triple Negative Breast Cancer Revealed by Multiplexed Ion Beam Imaging. Cell 2018. <https://doi.org/gd4wms>
10. Tibshirani R, Walther G: Cluster Validation by Prediction Strength. Journal of Computational and Graphical Statistics 2005. <https://doi.org/fvtcf4>
11. Schiebinger G, Shu J, Tabaka M, Jaenisch R, Regev A, Lander E, et al.: Optimal-Transport Analysis of Single-Cell Gene Expression Identifies Developmental Trajectories in Reprogramming. Cell 2019. <https://doi.org/gfwk5n>
12. Cang Z, Nie Q: Inferring spatial and signaling relationships between cells from single-cell transcriptomic data. Nature Communications 2020. <https://doi.org/gg9pf7>
13. Lundberg E, Borner GHH: Spatial proteomics: a powerful discovery tool for cell biology. Nature Reviews Molecular Cell Biology 2019. <https://doi.org/gft39v>
14. Dolédec S, Chessel D, Braak CJFT, Champely S: Matching species traits to environmental variables: a new three-table ordination method. Environmental and Ecological Statistics 1996. <https://doi.org/fhwz55>
15. Peres-Neto PR, Dray S, Braak CJFT: Linking trait variation to the environment: critical issues with community‐weighted mean correlation resolved by the fourth‐corner approach. Ecography 2016. <https://doi.org/10.1111/ecog.02302>
16. Griffiths JA, Scialdone A, Marioni J: Using single‐cell genomics to understand developmental processes and cell fate decisions. Molecular Systems Biology 2018. <https://doi.org/gdgbtq>
17. Lee HJ, Hore TA, Reik W: Reprogramming the Methylome: Erasing Memory and Creating Diversity. Cell Stem Cell 2014. <https://doi.org/f6f83c>
18. Clark SJ, Argelaguet R, Kapourani CA, et al.: scNMT-seq enables joint profiling of chromatin accessibility DNA methylation and transcription in single cells. Nat Commun. 2018 9, 781. <https://doi.org/10.1038/s41467-018-03149-4>
19. Argelaguet R, Clark SJ, Mohammed H, et al: Multi-omics profiling of mouse gastrulation at single-cell resolution. Nature 2019 576, 487–491. <https://doi.org/10.1038/s41586-019-1825-8>
20. Hanna CW, Demond H, Kelsey G: Epigenetic regulation in development: is the mouse a good model for the human? Human Reproduction Update 2018. <https://doi.org/gd3d4z>
21. Arora A, Olshen AB, Seshan VE, Shen R: Pan-cancer identification of clinically relevant genomic subtypes using outcome-weighted integrative clustering. Genome Med. 2020 12, 110. <https://doi.org/10.1186/s13073-020-00804-8>
22. Tenenhaus A, Philippe C, Guillemot V, Le-Cao KA, Grill J, Frouin J: Variable selection for generalized canonical correlation analysis. Biostatistics 2014. <https://doi.org/gg583d>
23. Rohart F, Gautier B, Singh A, Le-Cao KA: mixOmics: An R package for ‘omics feature selection and multiple data integration. PLOS Computational Biology 2017. <https://doi.org/gcj84s>
24. Tibshirani R, Hastie T: impute. Bioconductor 2017. <https://doi.org/gg9dds>
25. Atlasi Y, Stunnenberg H: The interplay of epigenetic marks during stem cell differentiation and development. Nat Rev Genet. 2017 18, 643–658. <https://doi.org/10.1038/nrg.2017.57>
26. Packer J, Trapnell C: Single-Cell Multi-omics: An Engine for New Quantitative Models of Gene Regulation. Trends in Genetics 2018 Volume 34 Issue 9 Pages 653-665 ISSN 0168-9525. <https://doi.org/10.1016/j.tig.2018.06.001>
27. Kempfer R, Pombo A: Methods for mapping 3D chromosome architecture. Nat Rev Genet. 2020 21, 207–226. <https://doi.org/10.1038/s41576-019-0195-2>
28. Schoenfelder S, Fraser P: Long-range enhancer–promoter contacts in gene expression control. Nat Rev Genet. 2019 485, 376-380.
29. Dixon JR, Selvaraj S, Yue F, et al.: Topological Domains in Mammalian Genomes Identified by Analysis of Chromatin Interactions. Nature 2012 485, 376-380. <https://doi.org/10.1038/nature11082>
30. Rao SSP, Huntley MH, Durand NC, Omer AD, Lander ES, Aiden EL: A 3D Map of the Human Genome at Kilobase Resolution Reveals Principles of Chromatin Looping. Cell 2014. <https://doi.org/10.1016/j.cell.2014.11.021>
31. Ibrahim DM, Mundlos S: The role of 3D chromatin domains in gene regulation: a multi-facetted view on genome organization. Curr Opin Genet Dev. 2020 Apr;61:1-8.<https://doi.org/10.1016/j.gde.2020.02.015>
32. Ing-Simmons E, Vaid R, Bing XY, et al: Independence of chromatin conformation and gene regulation during Drosophila dorsoventral patterning. Nat Genet. 2021 53, 487–499. <https://doi.org/10.1038/s41588-021-00799-x>
33. Schoenfelder S, et al.: The pluripotent regulatory circuitry connecting promoters to their long-range interacting elements. Genome Research 2015. <https://doi.org/10.1101/gr.185272.114>
34. Madrid-Mencía M,Raineri E, Cao TBN, Pancaldi V: Using GARDEN-NET and ChAseR to explore human haematopoietic 3D chromatin interaction networks. Nucleic Acids Research. 2020 Volume 48, Issue 8 Pages 4066–4080. <https://doi.org/10.1093/nar/gkaa159>
35. Javierre BM, Burren OS, Wilder SP, Wallace C, Spivakov M, Fraser P: Lineage-Specific Genome Architecture Links Enhancers and Non-coding Disease Variants to Target Gene Promoters. Cell. 2016 Volume 167 Issue 5 Pages 1369-1384.e19, ISSN 0092-8674. <https://doi.org/10.1016/j.cell.2016.09.037>.
36. Jung I, Schmitt A, Diao Y et al*:* A compendium of promoter-centered long-range chromatin interactions in the human genome. Nat Genet 2019 **51,**1442–1449. <https://doi.org/10.1038/s41588-019-0494-8>
37. Chovanec P, Collier AJ, Krueger C, et al.: Widespread reorganisation of pluripotent factor binding and gene regulatory interactions between human pluripotent states. Nat Commun. 2021 12, 2098. <https://doi.org/10.1038/s41467-021-22201-4>
38. Szabo Q, Jost D, Chang JM, et al: TADs are 3D structural units of higher-order chromosome organization in Drosophila. Sci Adv. 2018;4(2):eaar8082. <https://doi.org/10.1126/sciadv.aar8082>
39. Zhou T, Zhang R, Ma J: [The 3D Genome Structure of single-cells](https://www.annualreviews.org/doi/abs/10.1146/annurev-biodatasci-020121-084709). Annual Review of Biomedical Data Science 2021 4:1, 21-41.
40. Singh A, Shannon CP, Gautier B, Rohart F, Vacher M, Tebbutt SJ, Lê Cao KA: DIABLO: an integrative approach for identifying key molecular drivers from multi-omics assays. Bioinformatics 2019. <https://doi.org/ggpt9c>
41. Lê Cao KA, Rossouw D, Robert-Granié C, Besse P: A Sparse PLS for Variable Selection when Integrating Omics Data. Statistical Applications in Genetics and Molecular Biology 2008. <https://doi.org/cw7zft>
42. Shen H, Huang JZ: Sparse principal component analysis via regularized low rank matrix approximation. Journal of Multivariate Analysis 2008. [https://doi.org/b7x3cc \](https://doi.org/b7x3cc%20\)
43. Waaijenborg S, Verselewel de Witt Hamer PC, Zwinderman AH: Quantifying the Association between Gene Expressions and DNA-Markers by Penalized Canonical Correlation Analysis. Statistical Applications in Genetics and Molecular Biology 2018. <https://doi.org/bpzb68>
44. González I, Déjean S, Martin P, Baccini A: CCA : An R Package to Extend Canonical Correlation Analysis. Journal of Statistical Software 2008. <https://doi.org/gf4f5m>
45. González I, Déjean S, Martin PGP, Gonçalves O, Besse P, Baccini A: Highlighting Relationships Between Heterogeneous Biological Data Through Graphical Displays Based On Regularized Canonical Correlation Analysis. Journal of Biological Systems 2011. <https://doi.org/bmbjf5>
46. Witten DM, Tibshirani R, Hastie T: A penalized matrix decomposition, with applications to sparse principal components and canonical correlation analysis. Biostatistics 2009. <https://doi.org/fd4g54>
47. Parkhomenko E, Tritchler D, Beyene J:Sparse Canonical Correlation Analysis with Application to Genomic Data Integration. Statistical Applications in Genetics and Molecular Biology 2009. <https://doi.org/b7x4jb>
48. Soneson C, Lilljebjörn H, Fioretos T,Fontes M:Integrative analysis of gene expression and copy number alterations using canonical correlation analysis. BMC Bioinformatics 2010. <https://doi.org/dtxhsx>
49. Fertig EJ, Ren Q, Cheng H, Hatakeyama H, Dicker AP, Rodeck U, Considine M, Ochs MF, Chung CH: Gene expression signatures modulated by epidermal growth factor receptor activation and their relationship to cetuximab resistance in head and neck squamous cell carcinoma. BMC Genomics 2012. <https://doi.org/gb3fgp>
50. Li W, Zhang S, Liu CC, Zhou XJ: Identifying multi-layer gene regulatory modules from multi-dimensional genomic data. Bioinformatics 2012. <https://doi.org/f4d488>
51. Culhane AC, Perrière G, Higgins DG: Cross-platform comparison and visualisation of gene expression data using co-inertia analysis. BMC bioinformatics 2003. <https://doi.org/10.1186/1471-2105-4-59>
52. Witten D, Tibshirani RJ: Extensions of Sparse Canonical Correlation Analysis with Applications to Genomic Data. Statistical Applications in Genetics and Molecular Biology 2009. <https://doi.org/b45jtg>
53. Meng C, Basunia A, Peters B, Gholami AM, Kuster B, Culhane AC: MOGSA: Integrative Single Sample Gene-set Analysis of Multiple Omics Data. Molecular & Cellular Proteomics 2019. <https://doi.org/ggf3j3>
54. McCabe SD, Lin DY, Love MI: Consistency and overfitting of multi-omics methods on experimental data. Briefings in Bioinformatics 2020. <https://doi.org/gghpmf>
55. Kerr MK, Churchill GA: Bootstrapping cluster analysis: Assessing the reliability of conclusions from microarray experiments. Proceedings of the National Academy of Sciences 2001. <https://doi.org/cgpp6p>
56. Dudoit S, Fridlyand J: A prediction-based resampling method for estimating the number of clusters in a dataset. Genome Biology 2002. https://doi.org/10.1186/gb-2002-3-7-research0036
57. Haibe-Kains B, Desmedt C, Loi S, Culhane AC, Bontempi G, Quackenbush J, Sotiriou C: A Three-Gene Model to Robustly Identify Breast Cancer Molecular Subtypes. JNCI: Journal of the National Cancer Institute 2012. <https://doi.org/fzb27r>
58. Angel PW, Rajab N, Deng Y, Pacheco CM, Chen T, Lê Cao KA, Choi J, Wells CA: A simple, scalable approach to building a cross-platform transcriptome atlas. Plos Computational Biology 2020. <https://doi.org/10.1371/journal.pcbi.1008219>
59. Lavit C, Escoufier Y, Sabatier R, Traissac P: The ACT (STATIS method). Computational Statistics & Data Analysis 1994. <https://doi.org/c8xttz>
60. Sankaran K, Holmes SP: Multitable Methods for Microbiome Data Integration. Frontiers in Genetics 2019. <https://doi.org/gf8dqn>
61. Ramos M, Waldron L: MultiAssayExperiment. Bioconductor 2017. <https://doi.org/gg6p3d>
62. Ramos M, Schiffer L, Re A, Azhar R, Basunia A, Rodriguez C, Chan T, Chapman P, Davis SR, Gomez-Cabrero D, Waldron L, et al.: Software for the Integration of Multiomics Experiments in Bioconductor. Cancer Research 2017. <https://doi.org/gcj278>
63. Morgan M, Carlson M, Tenenbaum D, Arora S, Oberchain V, Morrell K, Shepherd L: ExperimentHub: Client to access ExperimentHub resources. Bioconductor 2017. <https://doi.org/gg6p3c>
64. Amezquita R, Lun A, Becht E, Carey V, Carpp L, Geistlinger L, Marini F, Rue-Albrecht K, Risso D, Soneson C, Waldron L, Pages H, Smith M, Huber W, Morgan M, Gottardo R, Hicks S: Orchestrating single-cell analysis with Bioconductor. Nature Methods 2020, 17, 137–145.

### Ramos M, Eckenrode K, Waldron L, Righelli D: SingleCellMultiModal: Integrating Multi-modal Single Cell Experiment datasets. Bioconductor 2021. <https://doi.org/gg95x5>
